# Effective Removal of Azithromycin from Aqueous Solution Using Coconut Husk Derived Boric Acid−Modified Biochar

**DOI:** 10.3390/toxics14100927

**Published:** 2026-10-20

**Authors:** Snigdha S. P. Mouna, Md. Al-Amin, Mohammad Mohinuzzaman, Md Shahadat Hossain, Abdulaziz Almuntashiri, M. M. Rahman, Mohammad Mahbub Kabir

**Affiliations:** 1 Department of Environmental Science & Disaster Management, Noakhali Science & Technology University, Noakhali 3814, Bangladesh; mouna0910@student.nstu.edu.bd (S.S.P.M.); alamin0914@student.nstu.edu.bd (M.A.-A.); 2 Department of Chemistry, Noakhali Science & Technology University, Noakhali 3814, Bangladesh; shahadat.chem@nstu.edu.bd; 3 Department of Civil and Environmental Engineering, Umm Al-Qura University, Makkah 24382, Saudi Arabia; aymuntashiri@uqu.edu.sa; 4 Bangladesh Council of Scientific and Industrial Research (BCSIR), Dhaka 1205, Bangladesh; mahbub.bcsir@yahoo.com; 5 School of Civil and Environmental Engineering, Faculty of Engineering and IT, University of Technology Sydney, NSW 2007, Australia

**Keywords:** azithromycin, biochar, adsorption, kinetics, isotherm

## Abstract

The occurrence of azithromycin in aquatic environments highlights the need for effective remediation approaches. In this study, three boric acid−modified coconut husk biochars prepared using nominal H_3_BO_3_ precursor concentrations of 0.1%, 2%, and 5% were evaluated for azithromycin adsorption from aqueous solution. The materials exhibited different adsorption performances under the investigated conditions, with 5% B−CB showing the highest Langmuir−predicted maximum adsorption capacity (*q_max_*) of 170.45 mg g^−1^, followed by 2% B−CB (158.84 mg g^−1^) and 0.1% B−CB (136.92 mg g^−1^). The equilibrium data were best statistically represented by the Langmuir model among the isotherm models evaluated. Kinetic analysis showed that the pseudo−second−order (PSO) model provided a better statistical description of the adsorption kinetics, with 5% B−CB showing the highest goodness−of−fit (R^2^ = 0.99), compared with 0.96 and 0.95 for 0.1% and 2% B−CB, respectively. The positive ΔH° values of 11.08, 6.74, and 6.19 kJ mol^−1^ for 0.1%, 2%, and 5% B−CB, respectively, indicated an endothermic adsorption process under the investigated conditions. FTIR analysis indicated qualitative changes in surface functionalities consistent with boric acid−modified coconut husk biochar following H_3_BO_3_−assisted modification; however, the actual boron content of the final biochars was not quantitatively determined. Furthermore, 5% B−CB retained measurable adsorption capacity over five adsorption–desorption cycles, and its fixed−bed performance was evaluated under the investigated operating conditions. Overall, the results indicate that azithromycin adsorption performance varied with the nominal H_3_BO_3_ precursor concentration, with 5% B−CB exhibiting the highest adsorption capacity among the materials investigated under the experimental conditions.

## 1. Introduction

Aquatic systems worldwide are increasingly affected by contaminants released from anthropogenic activities, including dyes, trace metals, and pharmaceutical residues. Among these contaminants, antibiotic residues have attracted particular attention because of their potential implications for environmental quality and public health. Antibiotics can enter aquatic environments through untreated or inadequately treated municipal wastewater, hospital effluents, pharmaceutical discharges, and other wastewater sources. Long−term exposure to this type of contamination can initiate the spread of drug−resistant bacteria along with the propagation of genetic resistance, which is undeniably a noteworthy issue [1].

Azithromycin is a macrolide antibiotic derived from erythromycin and is widely prescribed for the treatment of various bacterial infections. It is widely prescribed for the treatment of various bacterial infections, including pneumonia, intestinal infections, traveler’s diarrhea, otitis, and infections caused by *Streptococcus* species. Following use and disposal, azithromycin can enter wastewater and aquatic environments. Azithromycin remains in aquatic environments because its structure is stable and it breaks down only partially during wastewater treatment. This could be alarming for ecosystems and human health [2].

Conventional techniques for cleaning up wastewater include ion exchange [3], chemical−induced precipitation [4], photocatalytic degradation [5], solvent extraction [6], electrochemical techniques [7], oxidation based treatment methods [8], biological wastewater treatment [9], and adsorption [10] have demonstrated efficacy in eliminating toxic contaminants from contaminated water. However, the practical application of some of these approaches can be associated with relatively high operating or maintenance requirements, process complexity, and, depending on the treatment route, the possible formation of secondary products. On the other hand, because of its affordability, environmental friendliness, and potential for adsorbent regeneration and reuse, a straightforward, simple adsorption process has become popular in recent years [11]. Utilizing lignocellulosic agricultural wastes, specifically waste from tea [12], paddy husk [13], sugarcane bagasse [14], and sunflower husk has drawn more attention in recent years as a means of eliminating environmental pollutants. Additionally, these materials have a range of functional groups that facilitate biosorption, are abundant, and are sustainable. One such promising material is the husk of coconuts (*Cocos nucifera*), where cellulose, hemicellulose, and lignin constitute its major structural components [15]. Coconut husks, which comprise roughly 33–35% of the outer layer of coconuts, are an inexpensive and renewable biomass that can be efficiently transformed into adsorbents for harmful contaminants removal in wastewater [16].

The conversion of coconut husk into biochar provides a potential approach for both agricultural−waste management and wastewater treatment. Bangladesh, which ranked 12th globally in coconut production in 2021, has considerable potential for utilizing coconut husk as a locally available biomass resource for environmentally friendly wastewater treatment applications [17]. The production of biochar from coconut husk can therefore provide dual benefits by reducing agricultural waste while generating a potentially low−cost adsorbent for water treatment [15,18].

Modification of biochar using boric acid (H_3_BO_3_) has been investigated as a possible approach for altering the surface chemistry and adsorption characteristics of carbonaceous materials [19]. However, the extent and nature of such modifications depend on the preparation conditions and should be established through appropriate physicochemical characterization [19].

The present study therefore investigates the effect of increasing nominal H_3_BO_3_ precursor concentration on the physicochemical characteristics and azithromycin adsorption performance of coconut husk−derived biochar. Therefore, a thorough investigation of a variety of experimental variables including solution pH, equilibrium time, different azithromycin concentrations along with temperature, is intended to evaluate the adsorptive performance of the produced biochar. Additionally, it is intended to use equilibrium isotherm models to evaluate the adsorption performance, kinetic models to scrutinize the governing adsorption dynamics, and thermodynamic analysis to determine the nature and viability of the adsorption framework. This combined strategy not only aids in improving the adsorption conditions but also sheds light on the fundamental processes that control the modified biochar’s ability to remove azithromycin from wastewater.

## 2. Materials and Methods

### 2.1. Chemicals and Materials

This study employed analytical grade reagents, involving azithromycin, sodium hydroxide (NaOH, ≥99.0% purity), hydrochloric acid (HCl, 37% (*w*/*w*)), boric acid (H_3_BO_3_, ≥98% purity), potassium permanganate (KMnO_4_, 99% purity), and potassium carbonate (K_2_CO_3_, 99.995% purity). All chemicals, possessing approximately 98% purity, were procured from Merck GmbH, Darmstadt, Germany. Deionized water (DIW) was used as a solvent for analytical solution preparation during the experiments.

### 2.2. Fabrication of the Adsorbents

Coconut husks were obtained from the surrounding area and rigorously rinsed with DIW. Later, the coconut husks were fragmented into suitable portions and oven (Model: DSO−3000DF, Digisystem laboratory inc., Taiwan, China) dried for 2 h at 80 °C. Then the dried materials were crushed, and strained into multiple sizes varying from 0.015 to 0.25 mm. At that time, the synthesized powder underwent calcination in a muffle furnace (Model: JS MF−30T, Gongju City, Republic of Korea) at 600 °C for three consecutive periods. After that, the biochar samples were stabilized at room temperature before washing with deionized water as well as processed by oven drying at 105 °C for 24 h and this has been termed as “Pristine coconut husk biochar (P−CB)”. This biochar preparation method was adopted following the procedure reported by Dissanayaka et al. with slight modifications to suit the experimental requirements [20]. To fabricate biochar boric acid−modified coconut husk biochar, a method stated by Ates et al. was used [21]. To prepare 0.1, 2, and 5% (*w*/*v*) H_3_BO_3_ precursor solutions, 0.1, 2, and 5 g of boric acid were dissolved distinctly in 100 mL of DIW. Afterward, 3 g of coconut P−CB was mixed with 9 mL of the respective H_3_BO_3_ precursor solution and maintained under continuous contact for 1 day. The saturated samples were then dried and pyrolyzed at 600 °C for 3 h.

**Figure 1:**
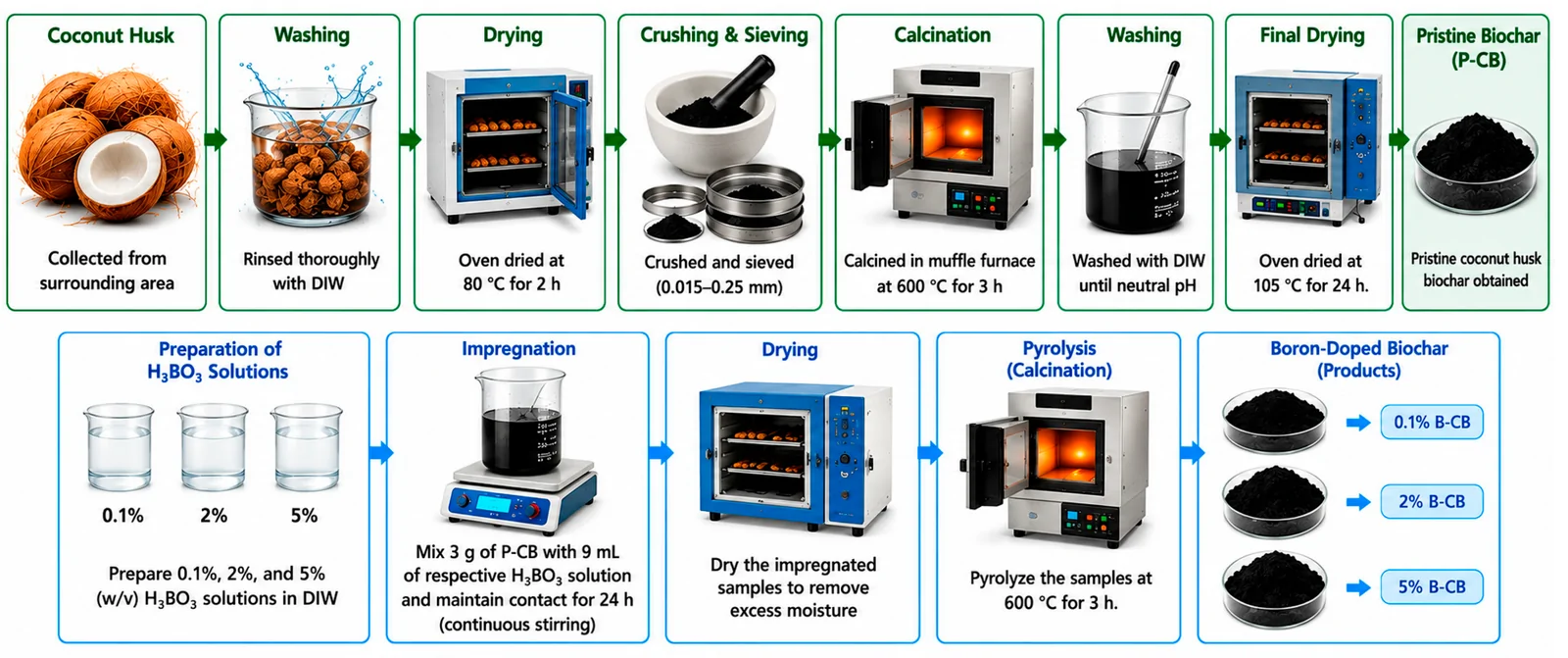
Schematic illustration of biosorbent synthesis.

For clarity, the resulting materials are designated as “0.1% Boric acid−modified coconut husk biochar (0.1% B−CB”), “2% Boric acid−modified coconut husk biochar (2% B−CB)”, and 5% Boric acid−modified coconut husk biochar (“5% B−CB”), where the percentages refer to the nominal concentration of the H_3_BO_3_ precursor solution and do not represent the measured boron content of the final biochar. The schematic synthesis of 0.1% B−CB, 2% B−CB along with 5% B−CB development is illustrated in Figure 1.

### 2.3. Characterization Profile of the Synthesized Adsorbents

FTIR analysis was performed to identify the chemical functionalities of the equipped adsorbents by using a PerkinElmer Spectrum 100, (Shelton, CT, USA). Also, for gaining insight into the morphological features and elemental distribution, FESEM−EDX analysis was performed via JEOL JSM−7610F (Tokyo, Germany). A UV−Visible spectrophotometer (Model: EMC−61PC−UV; EMC LAB; Duisburg, Germany) enabled the azithromycin level detection in both control and treated solutions. Additionally, the pH value in the aqueous solutions was observed with the aid of a digital pH meter (Model: Multi3510 IDS SET3, Germany) with microprocessor functionality, which was, as stated earlier, standardized with buffer solutions of recognized pH.

### 2.4. Adsorption Assessments of Azithromycin

The effectiveness of azithromycin removal was evaluated in batch adsorption experiments under controlled laboratory conditions with a rotary shaker (Model: JSSI−070C, JS Research Inc., Goyang-si, Republic of Korea). All batch experiments were conducted in a 100 mL conical flask containing 100 mL of azithromycin solution. The key objective of the experimental strategy was to inspect how adsorption performance was exaggerated by pH of the solution (2–10), contact time (5–60 min), adsorbent dosage (0.01–0.25 g), initial concentration of azithromycin (100–350 mg L^−1^), and temperature (25–60 °C). In the initial phase, a 100 mL conical flask was occupied with an azithromycin solution at optimal concentration and to preserve the preferred pH, either 0.1 M HCl or NaOH was added to the solution of azithromycin. A measured quantity of biosorbent was added to pH regulated experimental flasks, and the adsorption experiment was performed at 25 °C and 250 rpm.

Effect of contact time on azithromycin adsorption was studied in the range of 0–60 min. 100 mL of azithromycin solution with an initial concentration of 250 mg L^−1^ was used in each experiment with the initial pH of 7.0, adsorbent dosage of 0.1 g L^−1^, temperature of 25 °C and agitation speed of 250 rpm. Thus, the only varied parameter was the contact time and all other parameters of the experiment were kept constant.

The impact of solution pH on azithromycin adsorption was explored over the pH range of 2–10. For each experiment, 100 mL of azithromycin solution with an initial concentration of 250 mg L^−1^ was transferred into a 100 mL conical flask, and the pH was adjusted to the desired value using 0.1 M HCl or NaOH. The adsorbent dosage was maintained at 0.1 g. The experiments were conducted at 25 °C with an agitation speed of 250 rpm and a contact time of 40 min. All experimental parameters other than pH were kept constant.

The influence of initial azithromycin concentration was assessed over the range of 100–350 mg L^−1^. The azithromycin solution was 100 mL for each experiment. The initial pH value was maintained at 7.0, the adsorbent dose was 0.1 g L^−1^ (0.010 g/100 mL solution), the temperature was 25 °C, the contact time was 40 min and the agitation speed were 250 rpm. In this experimental series, only the initial azithromycin concentration was varied. All other parameters were constant.

The influence of temperature on azithromycin adsorption was studied in the temperature range of 25–60 °C. As for each experiment, 100 mL of the initial azithromycin solution with a concentration of 250 mg L^−1^ was taken, an adsorbent dose of 0.1 g L^−1^ was used, the initial pH was 7.0, the contact time was 40 min, and the agitation speed was 250 rpm. By varying the temperature from 25 to 60 °C, the influence of this factor on the adsorption capacity was evaluated, while other parameters remained the same.

The influence of the adsorbent dosage on the adsorption process was examined by altering the quantity of the adsorbent from 0.01 to 0.250 g per 100 mL of the azithromycin solution. In all experiments, the initial concentration of the antimicrobial substance was 250 mg L^−1^, the initial pH 7.0, contact time 40 min, temperature 25 °C, and the agitation speed 250 rpm, whereas the only variable was the adsorbent amount.

The centrifugation of the samples took place at 4000 rpm for 5 min after the specified period of contact time. A centrifuge (Model: SSU−173, Ambala, India) was used to obtain the mixture of adsorbent and aqueous from the centrifuge tube. The resultant mixture was then filtered with Whatman filter paper. The concentration of azithromycin in the desorbed solution was analyzed using a UV–visible spectrophotometer (Model: EMC−61 PC−UV; EM−CLAB, Duisburg, Germany) at 210 nm. The analytical wavelength was chosen by scanning the standard solution of azithromycin in the range of 200–400 nm and was in accordance with previously reported literature values [22,23]. A calibration curve was equipped using azithromycin standard solutions in the concentration range of 2–18 mg L^−1^ under the same analytical settings. A linear relationship between absorbance and azithromycin concentration was attained, with the calibration equation y = 0.0026695x − 0.0042929 and a coefficient of determination (R^2^) of 0.99926. DI water was used as the reagent blank, and blank absorbance was corrected before quantification. The residual standard deviation (Sy/x) of the calibration curve was 0.0004343. The limit of detection (LOD) and limit of quantification (LOQ) were calculated using LOD = 3.3σ/S and LOQ = 10σ/S, respectively, where σ represents the residual standard deviation of the calibration curve and S denotes the slope. The calculated LOD and LOQ were 0.537 and 1.627 mg L^−1^, respectively. Prior to UV–Vis analysis, the collected samples were diluted 20−fold with deionized water to ensure that the measured azithromycin concentrations fell within the validated calibration range of 2–18 mg L^−1^. The original azithromycin concentration was then calculated by applying the corresponding dilution factor.

However, azithromycin concentrations were determined from the standard calibration plot obtained under similar experimental conditions. The adsorption capacity and azithromycin removal rate of the adsorbents were calculated using two equations:

(1) $$q=(C_{i}-C_{f})\times \frac{V}{m}$$

(2) $$\mathrm{Extent}\;\mathrm{of}\;\mathrm{azithromycin}\;\mathrm{removal}\;(\%)=\frac{\left(C_{i}-C_{f}\right)}{C_{i}}\times 100$$

In this framework, *q* links to the adsorption performance of the synthesized adsorbents, stated in mg g^−1^, *V* implies the azithromycin sample volume (*L*), *C_i_* reflects the quantity of azithromycin in the medium before adsorption process (mg L^−1^), *C_f_* denotes the azithromycin concentration present in the solution at the end phase (mg L^−1^) and *m* symbolizes the amount of synthesized adsorbents (*g*) involved in the adsorption process.

### 2.5. Packed−Bed Column Adsorption Experiments

The adsorption study of azithromycin was conducted in a fixed−bed column packed with 150 g of 5% B−CB. The glass column had an internal diameter of 6.35 cm and a packed−bed height of 25.4 cm. A 0.5 mm glass wool layer and a 2 cm sand layer were placed at the bottom of the column to ensure uniform flow distribution and prevent the loss of adsorbent [24]. Azithromycin solutions with influent concentrations (*C_i_*) of 50 mg L^−1^ were adjusted to pH 7 and continuously passed through the packed bed using a peristaltic pump at a constant flow rate of 1 mL min^−1^. The column was operated at 25–30 °C, and effluent samples were collected periodically from the column outlet for up to 180 min. For the breakthrough experiment, the influent azithromycin concentration was 50 mg L^−1^. At a flow rate of 1 mL min^−1^, a total solution volume of 180 mL was passed through the column during the 180 min experimental period, corresponding to a total azithromycin input of 9.00 mg. Time zero (*t* = 0) was defined as the time when the azithromycin containing influent entered the packed bed. The residual azithromycin concentration in the collected effluent (*C_e_*) was determined by UV–Vis spectroscopy at approximately 210 nm following the analytical procedure described in Section 2.4.

The Supplementary Materials provide additional information on the fixed-bed column adsorption experiments, including the fitted parameters and goodness-of-fit statistics of the Thomas and Yoon–Nelson models (Supplementary Table S1).

### 2.6. Adsorption Modeling

#### 2.6.1. Kinetic Models of Adsorption

The kinetic study of adsorption behavior gives useful information on the rate at which a solute is adsorbed, which is important in determining the optimal contact time between the adsorbate and biosorbent. The kinetics of azithromycin adsorption onto the synthesized biochars were evaluated by three commonly used kinetic models pseudo−first−order (PFO), pseudo−second−order (PSO) and intraparticle diffusion (IPD) model [25,26]. Nonlinear regression was carried out using the original nonlinear forms of the PFO and PSO models and the contribution of the intraparticle diffusion was investigated using the IPD model. These formulas are as follows:

PFO kinetics

ln(*q_e_* − *q_t_*) = *lnq_e_* − *k*_1_*t* (3)

PSO kinetics

(4) $$\frac{t}{q_{t}}=\frac{1}{k_{2}q_{e^{2}}}+\frac{t}{q_{e}}$$

IPD model

*q_t_* = *k_id_t*^0.5^ + *C* (5)

In this context, *q_e_* (mg g^−1^) symbolizes the quantity of azithromycin adsorbed at equilibrium condition, while *q_t_* (mg g^−1^) refers to the amount of azithromycin adsorbed at time *t*, respectively [27]. *k*_1_ (min^−1^) is linked to the rate constant (min^−1^) of the PFO kinetics and stated as log(*q_e_* − *q_t_*) versus *t*. Furthermore, *k*_2_ (g mg^−1^min^−1^) represents the rate constant of the PSO adsorption (min^−1^) inferred from the plotted data *q_t_*/*t* versus *t*. Furthermore, k_id_ (mg g^−1^ min^−1^/^2^) represents the intraparticle diffusion rate constant and C (mg g^−1^) is the boundary layer constant that reflects the contribution of surface adsorption and the thickness of the boundary layer [28].

#### 2.6.2. Isotherm Models of Adsorption

In gain insight into the interaction pathways between the solute as well as the solution, along with the level of adsorbate−adsorption on the biosorbent surfaces, classical adsorption equilibrium sorption models, including the Langmuir along with the Freundlich models, were utilized in this current research [29]. These models are outlined in the following equations:

Freundlich isotherm model

(6) $$\log q_{e}=\log k_{f}+\frac{1}{n}\;\log C_{e}$$

Langmuir isotherm model

(7) $$\frac{1}{q_{e}}=\frac{1}{Q_{\max}}+\frac{1}{R_{L}\;Q_{\max}C_{e}}$$

(8) $$R_{L}=\frac{1}{1+K_{L}C_{i}}$$

Temkin isotherm model

*q_e_* = *B_T_*ln(*k_T_C_e_*) (9)

(10) $$B_{T}=\frac{\mathrm{RT}}{b_{T}}$$

Within this framework, 1/*n* and *k_f_* denotes the constants of Freundlich model that convey biosorption magnitude and solute uptake capability. In a similar manner, *q_e_* (mg g^−1^) is involved with solute uptake capacity and *C_i_* as well as *C_e_* (mg L^−1^) depicts the initial and equilibrium point concentration. Besides that, *R_L_* denotes dimensionless separation factor and *K_L_* symbolizes the Langmuir affinity constant (L mg^−1^) where *Q_max_* exemplifies the single−layer adsorption. In addition, *k_T_* (mg g^−1^) is the constant of Temkin equilibrium binding, *B_T_* represents the Temkin constant linked to the heat of adsorption, and *b_T_* (J mol^−1^) is the Temkin adsorption heat constant [27].

#### 2.6.3. Thermodynamic Models

Thermodynamic assessment of azithromycin adsorption was assessed by entropy ∆*S*^°^ (Jmol^−1^K^−1^), enthalpy ∆*H*^°^ (kJmol^−1^) and Gibbs free energy, ∆*G*^°^ (kJmol^−1^) acquired from a linear plot of *lnK_c_* versus 1/*T* [30].

∆*G*° = −*RTlnK_L_* (11)

(12) $$\ln k_{L}=\frac{∆S{}^{\circ}}{R}-\frac{∆H{}^{\circ}}{\mathrm{RT}}$$

In this section *K_L_* is attained from the solid as well as liquid segments from the azithromycin content ratio, *R* denotes the molar gas constant, and *T* stands for the solution’s absolute temperature (*K*).

The equilibrium distribution coefficient *K_L_* (L g^−1^) was calculated from the ratio of the equilibrium adsorption capacity (*q_e_*) to the equilibrium concentration (*C_e_*). Because qe/Ce has units of L g^−1^, it is treated here as an empirical distribution coefficient rather than a dimensionless standard−state equilibrium constant. Accordingly, the resulting thermodynamic quantities are interpreted within the convention adopted for this adsorption system. The standard Gibbs free energy change (Δ*G°*) was measured using equation no. 11. The standard enthalpy change (Δ*H°*) and standard entropy change (Δ*S°*) were attained from the slope and intercept, respectively, of the Van’t Hoff plot of *lnK_L_* versus *1*/*T*. The thermodynamic analysis was performed using equilibrium data attained at 298–333 K (25–60 °C). The linearity of the Van’t Hoff relationship was evaluated using the coefficient of determination (R^2^).

### 2.7. Regeneration and Reusability

Using azithromycin as the model pollutant, several adsorption–desorption cycles were used to assess the 5% B−CB’s reusability. Three desorbents were used to separate and regenerate the spent biochar following each adsorption: 50% ethanol (C_2_H_5_OH) in deionized water, 50% ethanol with 0.1 M NaCl, and 50% ethanol with 0.02 M NaOH [31,32,33] After one h of desorption at 25 °C with moderate agitation (150 rpm), the biomaterial was thoroughly rinsed with deionized water as well as dried at 60 to 80 °C. Under the same circumstances, the regenerated biochar was used again for adsorption cycles. Each cycle’s adsorption capacity (*q_e_*) and desorption capacity (*q_d_*) were computed, and the desorption efficiency (%) was ascertained as

(13) $$\mathrm{Desorption}\;\mathrm{rate}\;\left(\%\right)=\frac{q_{d}}{q_{e}}\times 100$$

### 2.8. Functions for Error Estimation

The regression fit (*R*^2^) values determined from the non−linear regression of the biosorption curves, along with the root mean square error (*RMSE*), were evaluated to establish the most effective model for interpreting the experimental data on azithromycin removal. The computational expressions adopted for the error values are as follows:

(14) $$\mathrm{RMSE}=\sqrt{\left(\frac{1}{n}\right)\sum_{i=1}^{n}{\left(q_{i.\exp}-q_{i.\mathrm{cal}}\right)}^{2}}$$

where *q_i_*_,*exp*_ (mg g^−1^) reflects the adsorption capacity determined through experimentation; *q_i_*_,*cal*_ (mg g^−1^) provides insights into the adsorption performance during the experiment. In this context, *n* refers to the number of recorded experimental results [34].

All experiments were conducted in triplicate. The adsorption models were fitted to the experimental data, and the goodness of fit was evaluated using the coefficient of determination (R^2^) and root mean square error (RMSE). Higher R^2^ and lower RMSE values were considered indicative of a better fit between the experimental data and the corresponding adsorption model. All calculations and model−fitting analyses were performed using GraphPad Prism (Version 8) and OriginPro 2021 software, as appropriate.

## 3. Results

### 3.1. Characterization of Adsorbent

**Figure 2:**
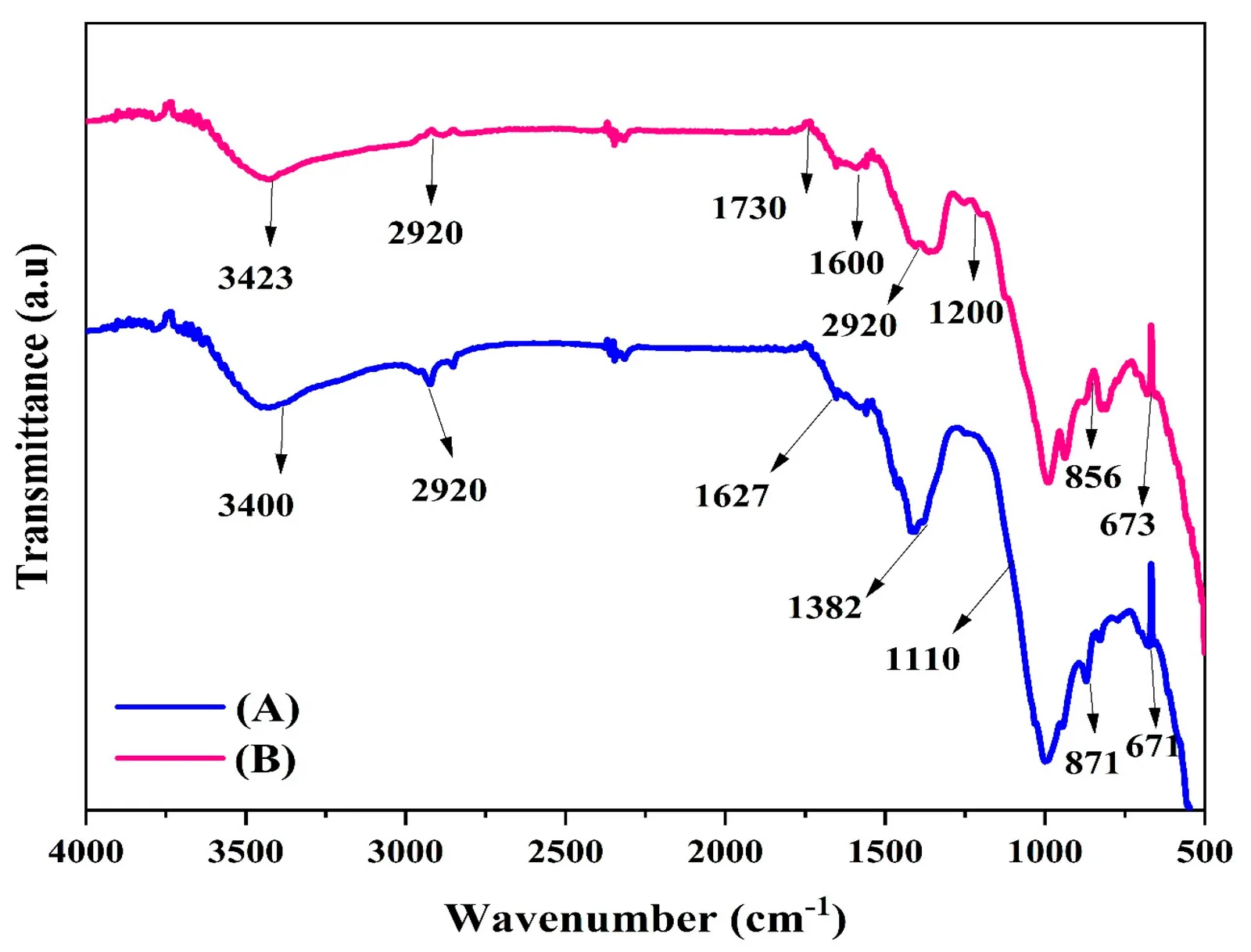
FTIR spectral changes in 5% B−CB due to azithromycin adsorption: (A) Before. (B) After.

The characteristic absorption bands before and after azithromycin adsorption were analyzed using FTIR, as shown in Figure 2. Hence, the FTIR analysis of 5% B−CB before adsorption is shown in Figure 2A and after sorption is outlined in Figure 2B. A broad absorption band at 3400 cm^−1^ before adsorption shifted to 3423 cm^−1^ after adsorption, corresponding to stretching vibrations indicating the existence of hydroxyl functionalities, which are likely associated with either water molecules or −OH groups attached to the material surface involved in hydrogen bonding. Both spectra exhibited a band at 2920 cm^−1^, which was indicative of the stretching mode of C–H bonds of aliphatic –CH_2_ groups, indicating the presence of residual organic matter or adsorbed azithromycin [35]. A characteristic vibration was observed at 1623 cm^−1^ before azithromycin adsorption and at 1600 cm^−1^ after azithromycin adsorption, associated with aromatic C=C bonds, indicating aromatic compound characteristics or graphitic structures in the biochar. On the contrary, a significant variation found at 1730 cm^−1^ after adsorption signifies the C=O bond stretching vibration from carbonyl functionalities present in the structure involving carboxylic acids or esters, potentially introduced or shifted due to adsorbate binding. Also, a significant band was found after the adsorption process at 1200 cm^−1^, reflecting C−O−C and C−O vibrational stretching possibly from esters or ether linkages. Another stretching at 1382 cm^−1^ before adsorption indicates C−H and −OH bending, which is associated with phenolic or alcoholic groups. A band near 1110 cm^−1^ was tentatively assigned to B–O stretching, providing qualitative evidence consistent with boron−containing surface species following H_3_BO_3_−assisted modification. Additionally, vibrational bands identified at approximately 871 and 856 cm^−1^ before and after adsorption, respectively, may be associated with B–C− or B–O–C−related vibrations [36,37]. Also, potential C−H bending of halogenated groups (C−Cl, C−Br) has been found at 671 cm^−1^ and 673 cm^−1^ pre−adsorption and post−adsorption stages, respectively. The changes at 1730 and 1200 cm^−1^ suggest alterations in carbonyl− and C–O−containing surface functionalities following adsorption. Shifts in the main peaks for O−H and C=C suggest hydroxyl and aromatic groups participated in the adsorption. The observed B–O and B–O–C−related bands provide qualitative evidence consistent with boron−containing surface species following H_3_BO_3_ treatment. These spectral variations suggest that azithromycin may interact with multiple surface functional groups on the adsorbent through hydrogen bonding and other possible non−covalent interactions. However, the observed FTIR spectral changes alone do not provide conclusive evidence of specific adsorption mechanisms, such as covalent bonding or ion exchange. Therefore, these interactions are considered plausible rather than definitively established [38].

**Figure 3:**
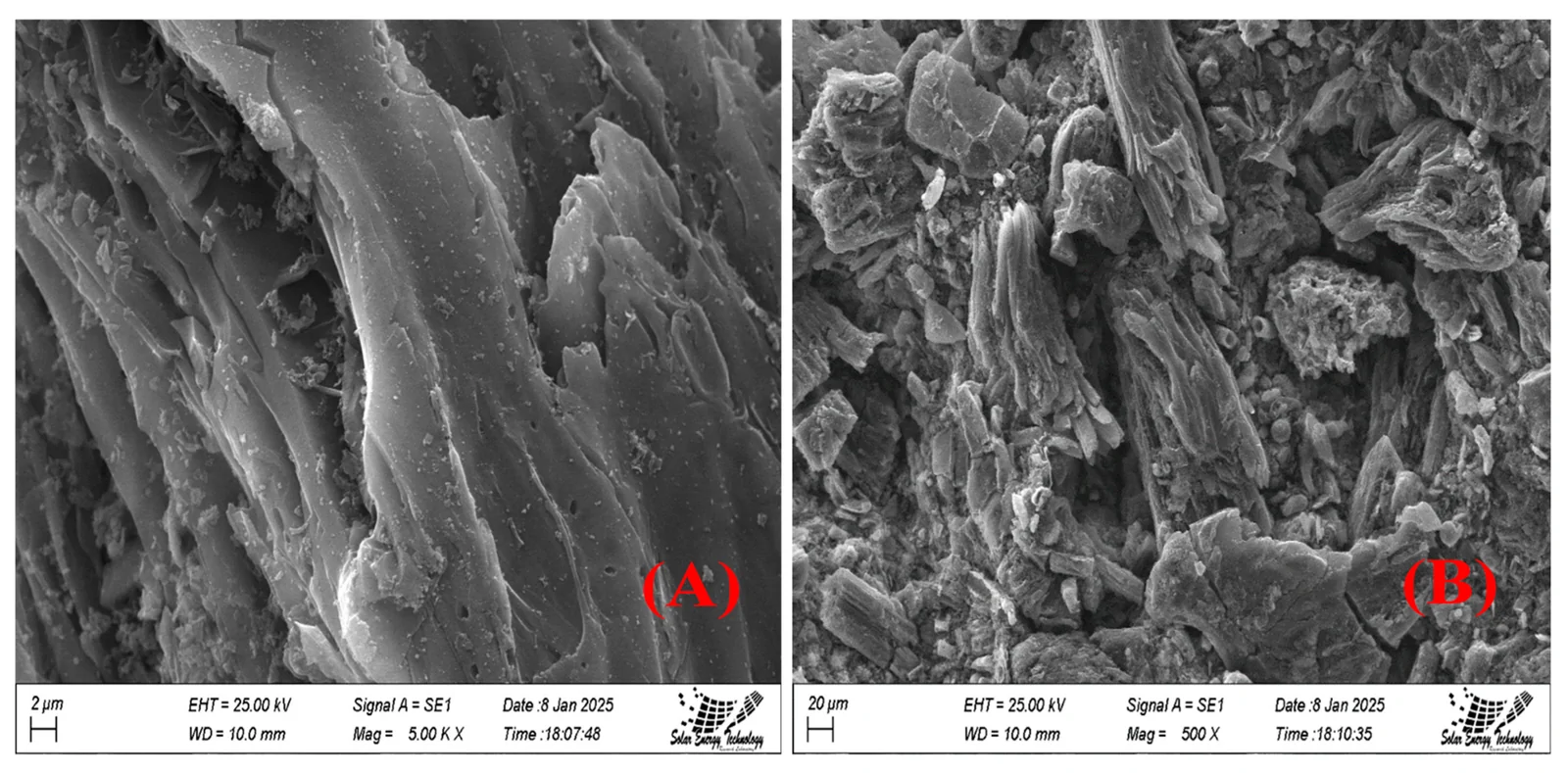
FESEM micrographs of (**A**) unmodified coconut husk (CH) biochar and (**B**) 5% boric acid−modified coconut husk biochar (5% B−CB) composite.

**Figure 4:**
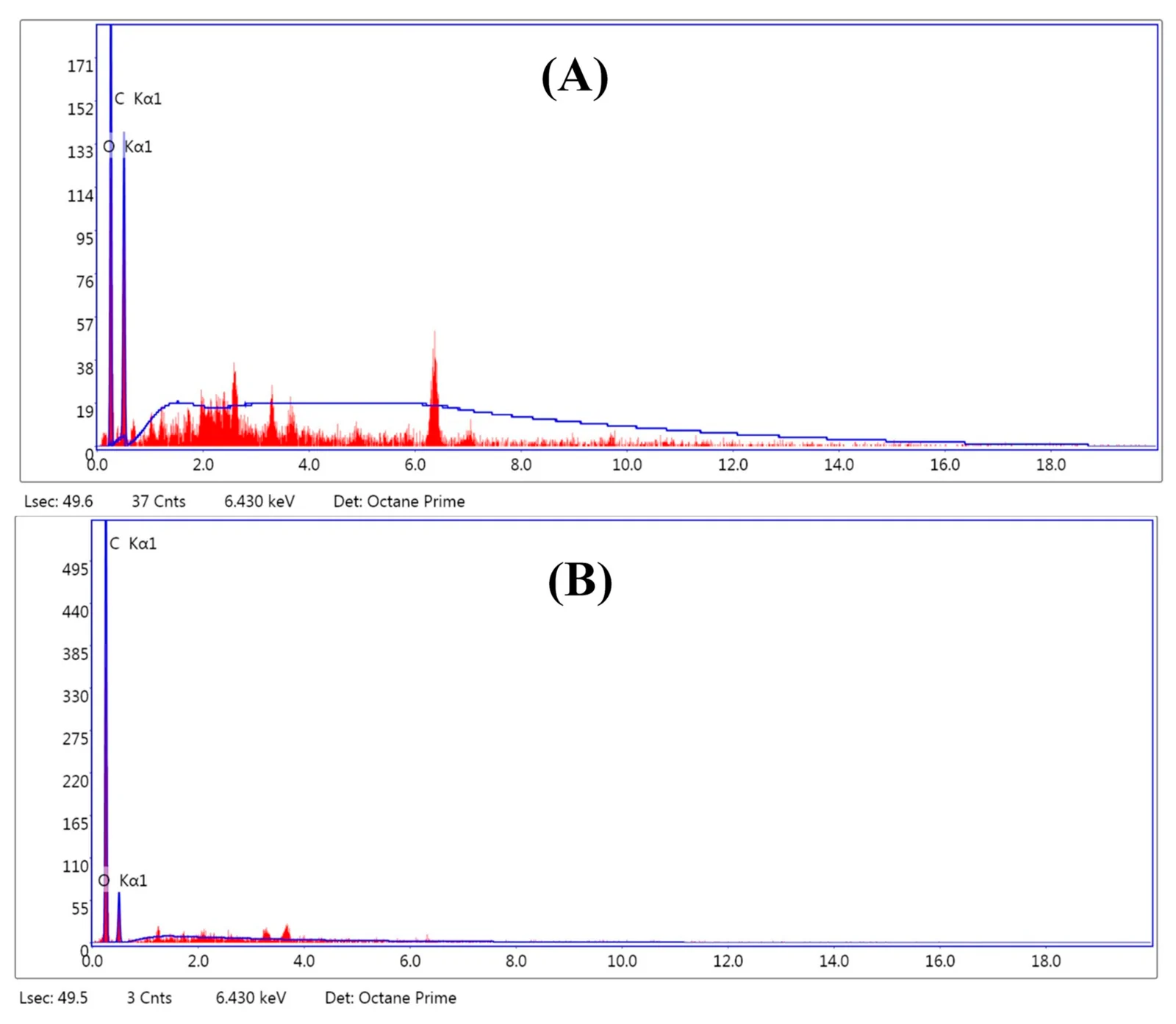
Elemental mapping by EDX of (**A**) Pristine Coconut husk Biochar (P−CB), and (**B**) 5% boric acid−modified coconut husk biochar (5% B−CB) composite.

FESEM micrographs of the synthesized unmodified CH biochar and 5% B−CB composite are shown in Figure 3. The findings demonstrated that unmodified CH biochar exhibited smooth and compact surface morphologies, with a low number of visible pores and minimal structural irregularity, signifying a relatively compact surface morphology with fewer visibly accessible surface features (Figure 3A). Furthermore, some layer−like formations are visible, approaching the collapsed plant cell walls. The morphology appears less fractured, indicating a denser and more solid texture. Additionally, the surface is relatively smooth and there are less visible voids, indicating a less developed apparent pore structure. Such morphological features may provide less visibly accessible sites for adsorbate interaction [39]. On the contrary, the surface morphology of 5% B−CB reveals a rough, irregular, and porous structure, which shows layered or sheet−like fragments with uneven surfaces, indicating the generation of graphitic carbon networks during pyrolysis (Figure 3B). The surface morphology of 5% B−CB was rougher and more irregular than that of P−CB, with visible spaces, cracks and surface irregularities. These are differences in the apparent surface morphology and may offer accessible areas for adsorbate−surface interactions. However, FESEM observations alone do not allow the quantitative determination of the variations in specific surface area, pore volume or pore size distribution. In addition, several sharp−edged particles and flaky or fibrous structures were observed, similar to the heterogeneous carbonaceous morphology of the biomass−derived biochar The FTIR bands tentatively assigned to B–O− and B–O–C−related vibrations provide qualitative indications of surface functionalities associated with boric acid−assisted modification [19]. The EDX analysis exhibited that the synthesized 5% B−CB was composed of 77.74% carbon and 22.26% oxygen in terms of weight, where raw coconut husk contains about 50.7% carbon content and 49.2% oxygen (Figure 4A,B).

**Figure 5:**
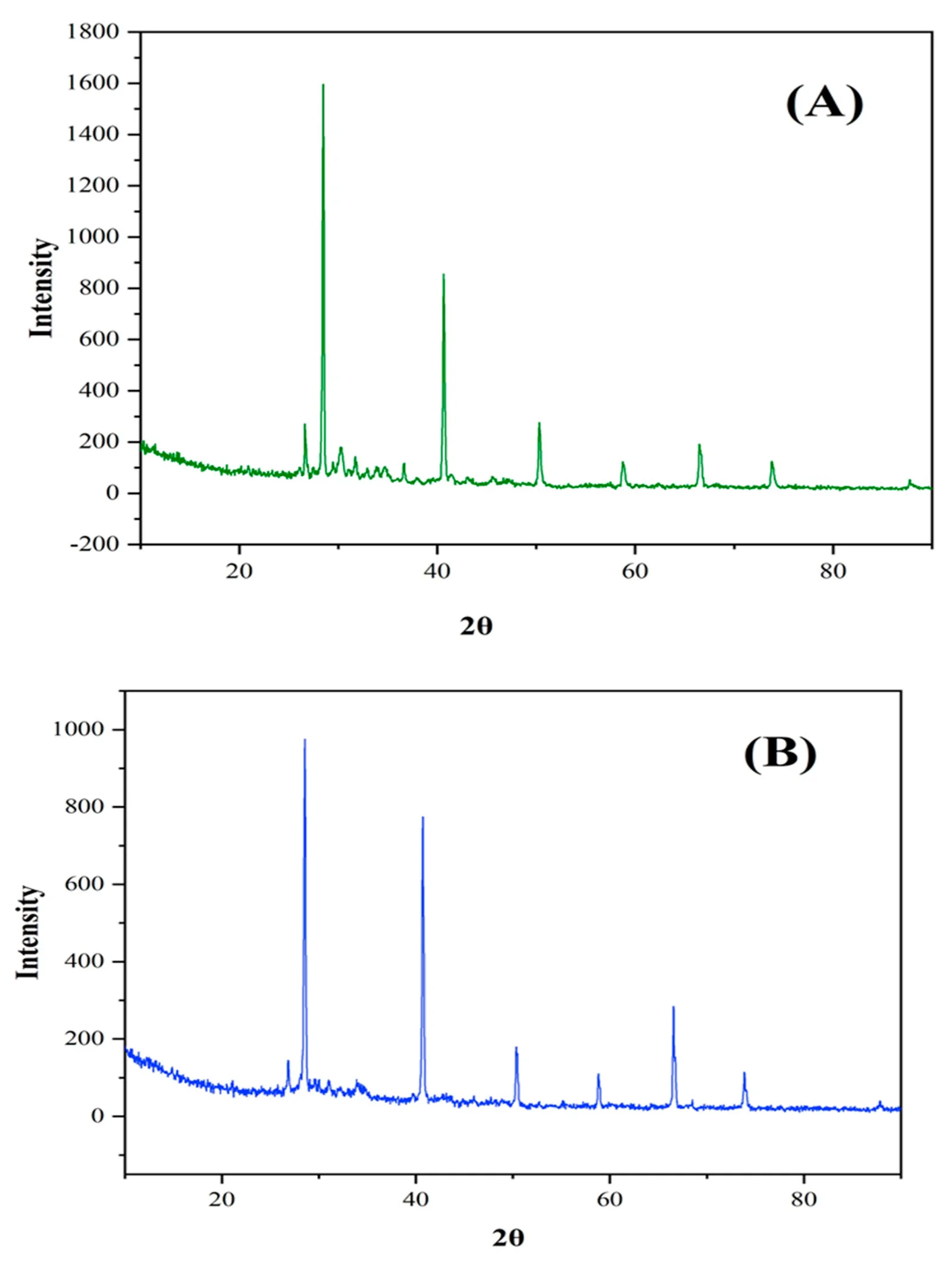
X-ray diffraction (XRD) pattern of (**A**) Pristine Coconut husk Biochar (P−CB), and (**B**) 5% boric acid−modified coconut husk biochar (5% B−CB) composite.

The XRD patterns of pristine coconut husk biochar (P−CB) and 5% boric acid−modified coconut husk biochar (5% B−CB) indicated that both materials were predominantly composed of amorphous carbon and silica phases (Figure 5A,B); however, significant differences emerged following boric acid modification. Both samples showed a broad diffraction peak that was linked to carbon (JCPDS card no. 01-075-0444), which means that the carbonaceous matrix was still the main phase. The broad profile is characteristic of a relatively disordered or poorly crystalline carbonaceous structure. Such a disordered carbonaceous structure may be associated with differences in surface characteristics; however, the porosity and surface area cannot be directly evaluated by XRD analysis. Both P−CB and 5% B−CB had diffraction peaks that matched those of silicon oxide (SiO_2_) (JCPDS card nos. 01-075-3165, 01-070-3316, 01-086-4010, and 00-046-1242). This was because coconut husk has a mineral composition that is naturally present. The silica framework of the raw biochar was preserved because these peaks stayed almost the same after modification. This means that the difference in adsorption performance between the two samples was not attributed to the silica framework.

Nevertheless, the XRD pattern of 5% B−CB exhibited noticeable differences from that of P−CB after boron modification. In particular, several additional weak and broad diffraction features were observed in the 5% B−CB pattern, which were tentatively assigned to H_3_BO_3_ and boron−containing carbide phases such as BC_7_ and B_4_C by comparison with the corresponding reference patterns (JCPDS Nos. 00-001-0767, 01-080-4613, and 00-001-1163). The presence of these weak reflections may indicate the formation of boric acid−modified coconut husk biochar during H_3_BO_3_−assisted pyrolysis. However, because the reflections are weak and/or broad, XRD alone does not provide sufficient evidence to unambiguously confirm the identity, abundance, or extent of incorporation of these phases within the carbon matrix. Therefore, these assignments should be regarded as tentative rather than definitive.

The observed changes in the diffraction pattern suggest that boron modification altered the structural characteristics of the biochar. Such modification may influence the local carbon structure and surface chemistry, including defect environments and oxygen−containing functional groups. However, the presence and specific nature of boron−related electronic defects cannot be established from the available XRD data alone. These changes could potentially generate or modify adsorption−active sites and thereby affect the interaction of the adsorbent with azithromycin. However, the specific contributions of boron species, surface defects, and textural properties to adsorption performance cannot be separated based on XRD results alone and require complementary surface−chemical and textural characterization.

Compared with P−CB, which predominantly exhibited broad amorphous carbonaceous features together with silica−related reflections, 5% B−CB showed additional weak reflections associated tentatively with boric acid−modified coconut husk biochar. Overall, the XRD results provide qualitative evidence of structural changes following boron modification and support the possibility that these changes were associated with the observed adsorption performance of 5% B−CB. Nevertheless, a direct causal relationship between the individual XRD features and the higher azithromycin adsorption capacity cannot be established solely from XRD analysis. Previous studies have similarly reported that boron modification can alter the surface polarity, adsorption behavior, and electron−transfer characteristics of carbon−based materials [28,40,41].

### 3.2. Optimization Study on the Adsorption Parameters of Azithromycin

Recent research has prioritized the creation of novel, cost−effective adsorbents for removing toxic pollutants from different environmental media. This research investigates boric acid modified biochar to elucidate the elimination of azithromycin from aqueous solution.

Table 1 summarizes the reported adsorption capacities of different adsorbents for azithromycin elimination under their respective experimental settings. Though the adsorption capacities stated in the literature cannot be considered directly comparable because of variations in initial azithromycin concentration, adsorbent dosage, pH, temperature, and contact time, the results provide a useful comparative perspective on the performance of the developed adsorbents. In the present investigation, pristine coconut−husk biochar (P−CB) exhibited an adsorption capacity of 92 mg g^−1^, whereas the boric acid−modified biochars exhibited higher capacities of 121, 136.1, and 141 mg g^−1^ for 0.1% B−CB, 2% B−CB, and 5% B−CB, respectively. Among the synthesized adsorbents, 5% B−CB exhibited the highest adsorption capacity (141 mg g^−1^), followed by 2% B−CB (136.1 mg g^−1^) and 0.1% B−CB (121 mg g^−1^). Under the experimental conditions of this study, the progressive increase in adsorption capacity with increasing nominal H_3_BO_3_ precursor concentration suggests that H_3_BO_3_−assisted modification contributed positively to the adsorption performance of coconut−husk biochar. However, this comparison should be interpreted in the context of the specific experimental conditions applied to each adsorbent.

Although the adsorption capacity increased with increasing nominal H_3_BO_3_ precursor concentration, the improvement was not proportional to the increase in nominal H_3_BO_3_ precursor concentration. The adsorption capacity increased from 121 mg g^−1^ for 0.1% B−CB to 136.1 mg g^−1^ for 2% B−CB and 141 mg g^−1^ for 5% B−CB. Thus, the increase from 2% to 5% B−CB was relatively smaller than that observed from 0.1% to 2% B−CB, indicating a diminishing improvement at higher nominal H_3_BO_3_ precursor concentrations. This behavior may be associated with the progressive modification of surface functional groups and the increasing accessibility of adsorption sites, while increasing the nominal H_3_BO_3_ precursor concentration may not proportionally generate new effective adsorption sites. Because BET characterization was not performed, it is not possible to conclusively determine whether changes in surface area or pore structure contributed to this trend. Therefore, the enhanced adsorption performance of 5% B−CB is more cautiously attributed to the combined effects of surface chemical modification and accessibility of adsorption sites [42].

**Figure 6:**
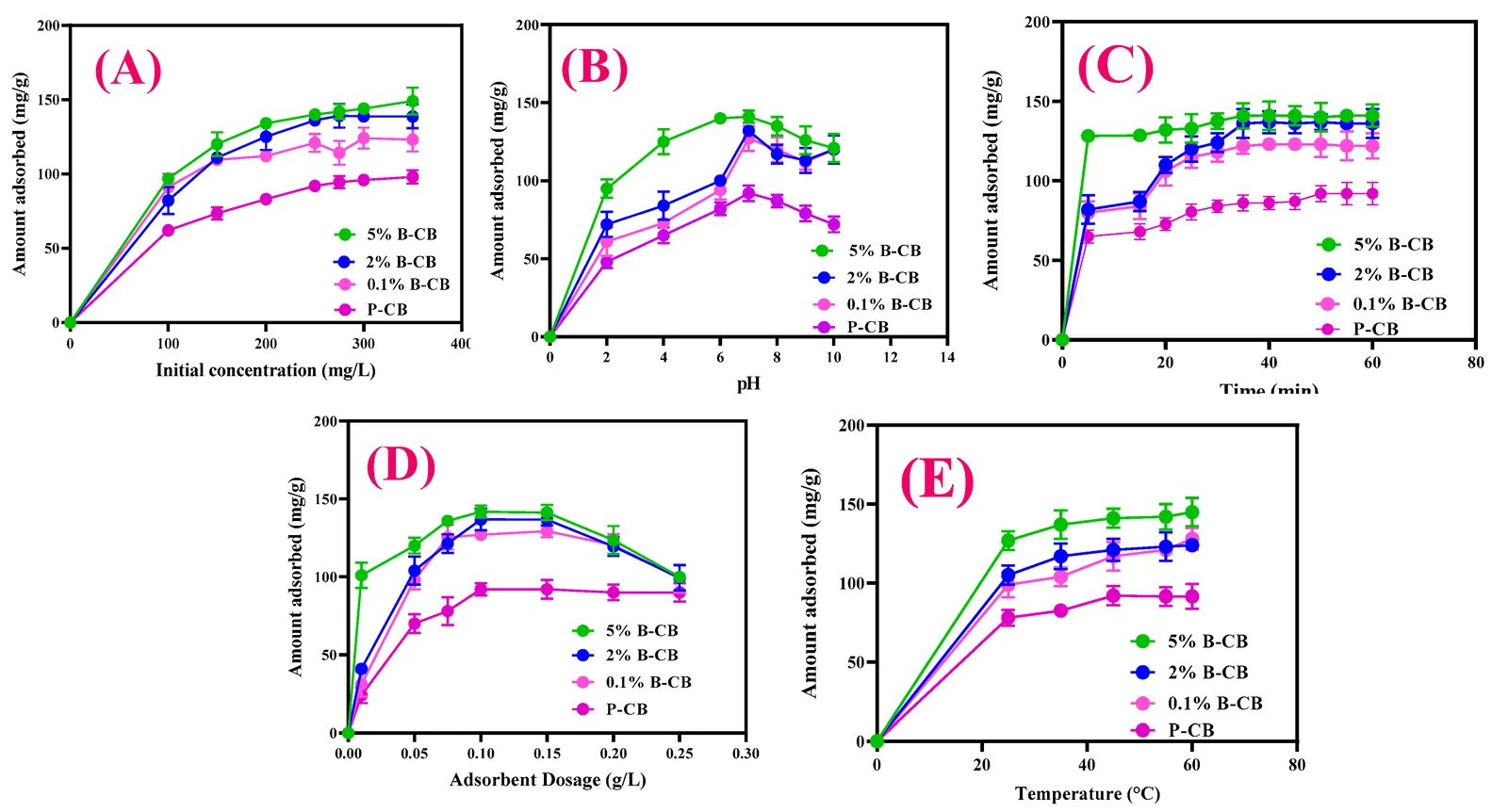
Investigation of key operational parameters affecting the efficiency of azithromycin removal: (**A**) concentration, (**B**) solution pH, (**C**) contact time, (**D**) adsorbent dosage, and (**E**) temperature. Error bars represent standard deviation (n = 3).

Figure 6A shows that the uptake of azithromycin with 0.1, 2, and 5% B−CB increased as the concentration of azithromycin went up. The concentrations of azithromycin ranged from 100 to 350 mg L^−1^, using an adsorbent dosage of 0.1 g L^−1^, an contact time of 35 min, a temperature of 25 °C, and pH was adjusted to 7. As initial azithromycin concentrations increase, adsorption capacity also goes up slowly. Hence, the peak adsorption capacity was detected with the initial concentration set to 250 mg L^−1^, yielding adsorption efficacies of 141 mg g^−1^, 121 mg g^−1^, and 136 mg g^−1^ for 5% B−CB, 2% B−CB, and 0.1% B−CB, correspondingly. Under the same experimental conditions, pristine coconut−husk biochar (P−CB) exhibited a lower adsorption capacity of 92 mg g^−1^, confirming the improved adsorption performance of the boric acid−modified biochars. The adsorption percentage decreased when the concentration of azithromycin reached its ultimate at 350 mg L^−1^. This reduction is linked with the inadequate reactive spots on the biosorbent’s surface, which become occupied as azithromycin levels increase [43].

The solution pH significantly influenced azithromycin adsorption because it affects both the surface chemistry of the biosorbent and the ionization state of the adsorbate, thereby regulating their interfacial interactions. Changes in pH can alter the protonation and deprotonation of surface functional groups and consequently modify the surface charge of the adsorbent. As shown in Figure 6B, highly acidic conditions (pH 2–4) adversely affected the adsorption performance. The adsorption capacities were 95–125 mg g^−1^ for 5% B−CB, 72–84 mg g^−1^ for 2% B−CB, and 61–73 mg g^−1^ for 0.1% B−CB. In comparison, pristine coconut−husk biochar (P−CB) exhibited lower adsorption capacities of approximately 48–65 mg g^−1^ under acidic conditions (pH 2–4). This lower adsorption at strongly acidic pH can be attributed mainly to the high concentration of H^+^ ions, which may compete with azithromycin for available active sites and alter the protonation state of surface functional groups.

When the solution pH increased from slightly acidic to near−neutral conditions (pH 6–7), the adsorption capacity increased and reached a maximum at pH 7, with values of 141 mg g^−1^ for 5% B−CB, 132 mg g^−1^ for 2% B−CB, and 127 mg g^−1^ for 0.1% B−CB. Under the same conditions, P−CB showed a lower adsorption capacity of 92 mg g^−1^ at pH 7, further demonstrating the enhanced adsorption performance achieved through boric acid modification. This enhanced adsorption suggests more favorable interactions between azithromycin and the available adsorption sites on B−CB under near−neutral conditions. The observed behavior can be further explained by considering the acid–base properties of azithromycin and the surface charge of B−CB. Azithromycin is a weak dibasic compound with reported pKa values of approximately 8.74 and 9.45 [44]. Thus, at pH values below its pKa range, protonated/cationic forms of azithromycin are favored, whereas increasing pH progressively decreases its degree of protonation.

The observed pH dependence may reflect the combined effects of azithromycin speciation and pH−dependent changes in surface functionalities. Because pHpzc and zeta−potential measurements were not performed, the contribution of surface charge to the observed adsorption behavior could not be directly quantified. At near−neutral pH, protonated forms of azithromycin are expected to remain prevalent based on its reported pKa values, while the ionization state of oxygen− and boron−containing surface functional groups may also change with pH. These conditions may favor interactions between azithromycin and available surface functionalities, potentially involving hydrogen bonding and other interfacial interactions. Electrostatic contributions are also possible based on azithromycin speciation; however, they could not be directly verified because surface−charge measurements were not performed. However, the specific contribution of surface charge could not be directly quantified in the present study because zeta−potential/pHpzc measurements were not performed. This combination likely contributed to the maximum adsorption observed at pH 7.

At higher pH (8–10), the adsorption capacity gradually decreased. This behavior may be associated with the progressive deprotonation of azithromycin as the solution pH approaches and exceeds its reported pKa range, which can reduce the fraction of positively charged azithromycin species available for favorable electrostatic interactions with ionized surface functionalities. Changes in the protonation state of surface functional groups and possible competition from OH^−^ ions for polar adsorption sites may also contribute to the observed decrease. Because surface charge was not directly measured, these effects are considered possible contributors rather than experimentally verified electrostatic mechanisms [38].

Hence, the adsorption of azithromycin by 0.1%, 2% and 5% B−CB exhibited a bell−shaped dependence on solution pH, with the highest adsorption observed under near−neutral conditions (pH 6–7). The results indicate that the optimum adsorption performance may reflect a balance between azithromycin speciation and pH−dependent changes in surface functionalities.

This study assessed the role of contact period (5–60 min) at steady thermal conditions of 25 °C on the sorption capability of 0.1% B−CB, 2% B−CB, and 5% B−CB for azithromycin removal, as depicted in Figure 6C. During the initial phase (0–5 min), a swift enhancement in adsorption capacity was noted, reaching 80 mg g^−1^, 82 mg g^−1^, and 128.2 mg g^−1^ for 0.1% B−CB, 2% B−CB, and 5% B−CB, respectively. In comparison, pristine coconut−husk biochar (P−CB) exhibited an adsorption capacity of 65 mg g^−1^ at 5 min, which was lower than those of all boric acid−modified biochars under the same conditions. This initial surge reflects the extensive presence of available surface binding sites and a steep concentration gradient that accelerated mass transfer. The adsorption rate decelerated with increasing contact time, with the capabilities expanding to 115 mg g^−1^, 120 mg g^−1^, and 132.9 mg g^−1^ by 25 min and further to 118 mg g^−1^, 124 mg g^−1^, and 137.5 mg g^−1^ by 30 min for 0.1% B−CB, 2% B−CB, and 5% B−CB, correspondingly. At 30 min, P−CB showed a lower adsorption capacity of approximately 80 mg g^−1^, further demonstrating the enhanced azithromycin uptake of the boric acid−modified biochars. The adsorption leveled off after about half an hour, signifying it was nearing equilibrium. The adsorption capacities remained comparatively steady between 40 and 60 min, reaching approximately 123 mg g^−1^ for 0.1% B−CB, 137 mg g^−1^ for 2% B−CB, and 141 mg g^−1^ for 5% B−CB, whereas P−CB reached approximately 86–90 mg g^−1^ during the same period. The adsorption capacity increased rapidly during the initial stage and gradually approached equilibrium, likely due to the progressive occupation of available adsorption sites. Although the pseudo−second−order (PSO) model provided a good fit to the experimental data, this result alone does not confirm chemisorption as the dominant adsorption mechanism. Further spectroscopic and surface−chemical analyses are required to identify the specific interactions involved in azithromycin adsorption [45]. The quick reach of equilibrium demonstrates the effectiveness of the boric acid−modified biochar that was synthesized, particularly 5% B−CB, which had the highest adsorption capacity of all the samples that were tested. Such performance indicated the potential of 5% B−CB as a viable adsorbent for practical water treatment applications targeting pharmaceutical contaminants like azithromycin [46,47].

**Table 1:** Evaluation of the performance of 5% B−CB compared to other adsorbents in toxic pollutant removal.

Adsorbent	Initial Conc. (mg L^−1^)	Adsorbent Dose (g L^−1^)	pH	Temperature (°Celsius)	Contact Time (min)	Capacity (mg g^−1^)	References
Alkali−treated water hyacinth powder	50–2500	0.125–1.125	2–10	25	0–60	247	[48]
Pristine water hyacinth powder	50–2500	0.125–1.125	2–10	25	0–60	136	[48]
Magnetic activated carbon prepared from *Dalbergia Sissoo*	80	0.1	6	25–60	120	41	[49]
ZnO Nanoparticle	110	0.05	2	25	−	116	[50]
FAU−1 Zelolites	0.1	0.01	6.5	−	0–360	7.5	[51]
FAU−2 Zelolites	0.1	0.01	8.7	−	0–360	6	[51]
Natural Clinoplite	30–400	5	11	35	−	28	[28]
Natural clinoptilolite−Triton	30–400	5	11	35	−	44	[28]
Modified −Nano Diatomite	20–100	0.5–2.5	3–11	25	60	91	[52]
Natural clinoptilolite−Tween	30–400	5	11	35	−	36	[28]
MgAl−LDH/Biochar composite	−	−	12	40	20	406	[53]
RPS	50–300	0.01–0.15	2–11	25	0–60	186	[54]
PS−LDHs	50–300	0.01–0.15	2–11	35	0–60	330	[54]
P−CB	250	0.01–0.25	2–10	25–60	0–60	92	This study
0.1% B−CB	250	0.01–0.25	2–10	25–60	0–60	121	This study
2% B−CB	250	0.01–0.25	2–10	25–60	0–60	136.1	This study
5% B−CB	250	0.01–0.25	2–10	25–60	0–60	141	This study

The influence of varying dosage of adsorbent (0–0.25 g) on azithromycin adsorption performance was evaluated as 0.1% B−CB, 2% B−CB, and 5% B−CB biochar adsorbents and shown in Figure 6D. The results revealed a typical dose−dependent trend, as illustrated by the data. At relatively low dosage (0.01 g), the adsorption capacity rapidly increased, reaching 101 mg g^−1^ for 5% B−CB, 41 mg g^−1^ for 2% B−CB, and 31 mg g^−1^ for 0.1% B−CB. Under the same settings, pristine coconut−husk biochar (P−CB) displayed a lower adsorption capacity of approximately 24 mg g^−1^. This initial increase is likely due to more adsorbate molecules relative to available adsorption sites, which boosts the chance for interaction. The adsorption capacities peaked at 141.8 mg g^−1^ for 5% B−CB, 136.8 mg g^−1^ for 2% B−CB, and 129.3 mg g^−1^ for 0.1% B−CB when the dosage was raised to 0.075–0.15 g. In assessment, P−CB showed lower adsorption capacities of almost 78–85 mg g^−1^ within the same dosage range, further representing the enhanced adsorption performance of the boric acid−modified biochars. Therefore, the observed optimum dosage may reflect a balance between the amount of azithromycin available in solution and the number of accessible adsorption sites. The adsorption capacity of every sample decreased at a dose of 0.15 g. The resulting adsorption efficiencies for 5% B−CB, 2% B−CB, and 0.1% B−CB were 123.6 mg g^−1^, 119.5 mg g^−1^, and 120.5 mg g^−1^, while P−CB exhibited about 78 mg g^−1^ correspondingly, while a slightly increased dosage (0.2 g) was studied. When the dosage was further increased to 0.25 g, the adsorption capacities decreased to 99.96 mg g^−1^, 99.4 mg g^−1^, and 98.92 mg g^−1^ for 5% B−CB, 2% B−CB, and 0.1% B−CB, respectively, compared with about 70 mg g^−1^ for P−CB. At higher adsorbent dosages, particle aggregation or overlap of adsorption sites may reduce the accessibility of some adsorption sites and consequently decrease the adsorption capacity per unit mass [38]. Hanafiah et al. [55] observed close results when studying how well coconut husk adsorbed nickel and chromium. Overall, 5% B−CB exhibited comparatively higher adsorption capacities across the investigated dosage range. The data show that, increased adsorbent dosages improve removal efficiency, very high dosages might lower how well each unit adsorbs because the locations are full, which makes the surface less available [31].

The influence of temperature in adsorption study of 0.1, 2, and 5% B−CB were analytically estimated over the temperature range between 25 °C and 60 °C. The findings disclosed the thermally responsive features of the adsorption method by examining a positive association between temperature and adsorption capabilities for all synthesized biosorbents (Figure 6E). At 25 °C, the adsorption capacities were 127, 105, and 99 mg g^−1^ for 5%, 2%, and 0.1% B−CB, respectively. Under the same conditions, pristine coconut−husk biochar (P−CB) exhibited a lower adsorption capacity of approximately 78 mg g^−1^. This implies that increasing temperatures increases the vibrational energy of azithromycin molecules, thereby promoting their diffusion and attachment onto available active sites of the adsorbents. The adsorption performance improved further with increases to 60 °C, peaking at 145 mg g^−1^ (5% B−CB), 124 mg g^−1^ (2% B−CB), and 128 mg g^−1^ (0.1% B−CB). In comparison, P−CB exhibited an adsorption capacity of approximately 91 mg g^−1^ at 60 °C, remaining lower than those of all boric acid−modified biochars. This steady rise attests to the endothermic nature of azithromycin adsorption. Stronger interactions with azithromycin molecules may be made possible by higher thermal conditions, which may also make functional sites (such as hydroxyls, amines, and carboxyls) at the surfaces of the biochar more accessible and reactive. Rahim et al. [56] looked at a study that used a variety of chemicals, for example NaOH, NaOCl, H_2_O_2_, along with HNO_3_, to treat charred and desiccated coconut waste [56]. They found similar results. The consistently superior performance of 5% B−CB compared with P−CB and the other boric acid−modified biochars suggests that boron modification may have contributed to improved surface functionality and adsorption−site availability. However, because specific surface−area and surface−charge measurements were not performed, these contributions cannot be quantitatively separated in the present study. The enhanced adsorption of 0.1% B−CB at elevated temperatures further indicates that even a lower level of boron modification can improve azithromycin uptake under thermally favorable conditions [42].

### 3.3. Adsorption Kinetics, Isotherm, and Thermodynamics

#### 3.3.1. Kinetic Models

**Figure 7:**
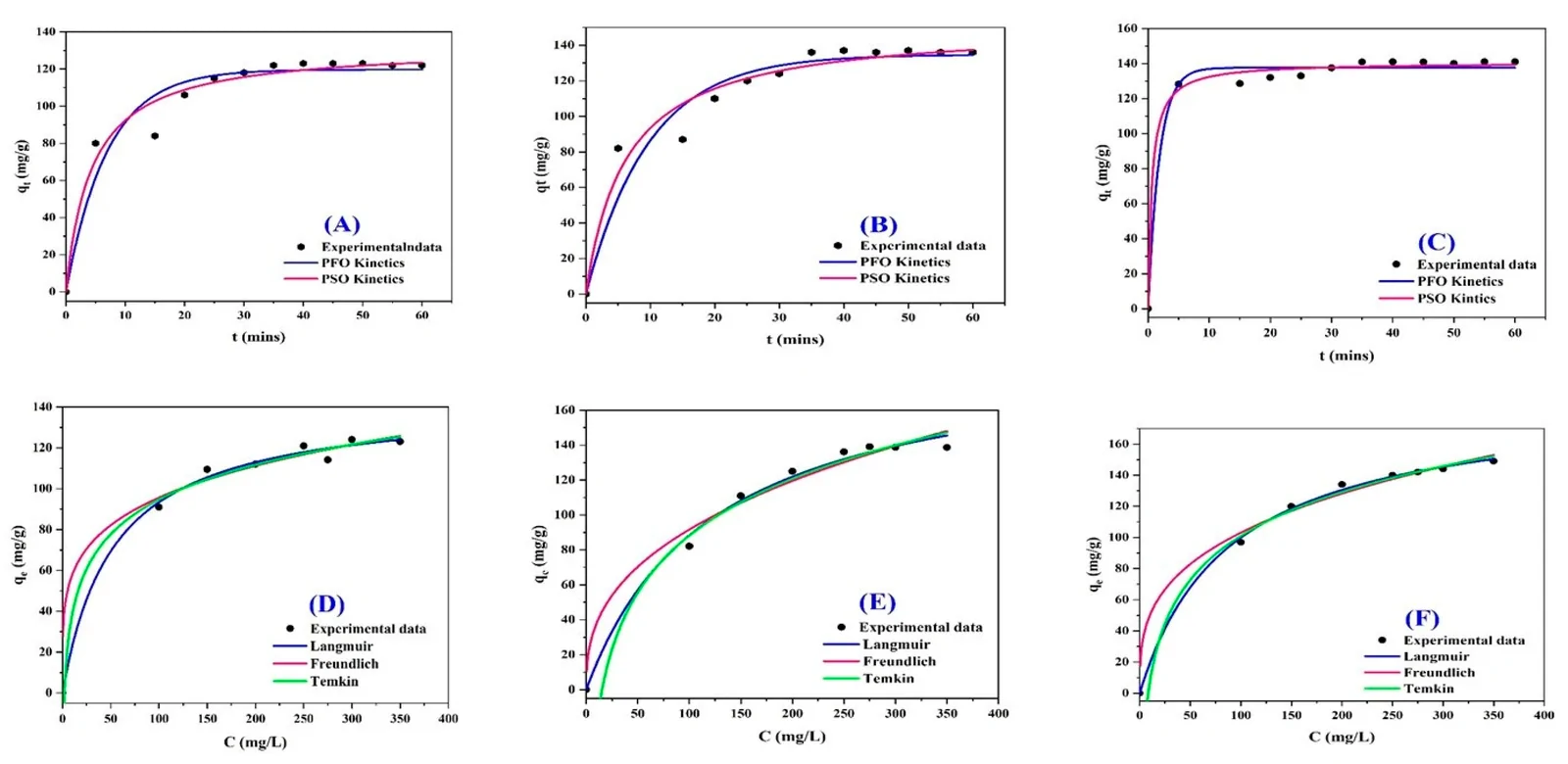
Time−dependent adsorption profile of azithromycin onto (**A**) 0.1% B−CB, (**B**) 2% B−CB, (**C**) 5% B−CB and Fitting of isotherm models for azithromycin adsorption onto (**D**) 0.1% B−CB, (**E**) 2% B−CB, (**F**) 5% B−CB.

The adsorption kinetic characteristics of azithromycin on 0.1, 2, and 5% B−CB were assessed via the PFO as well as PSO kinetic models, as illustrated in Figure 7A–C, while the fitted kinetic parameters were tabulated in Table 2. For all adsorbents, the adsorption capacity experienced a rapid rise during the preliminary phase and gradually attained equilibrium as contact time increased. In addition, the investigational equilibrium adsorption capabilities (qe,_exp_) were 121 mg g^−1^, 136.1 mg g^−1^, and 141 mg g^−1^ for 0.1, 2, and 5% B−CB, individually, demonstrating that boric acid modification improved the biosorption performance of the adsorbent.

The adsorption kinetic studies stated that both PFO and PSO kinetic models sufficiently explained the adsorption characteristics; nevertheless, the PSO kinetic model mostly exhibited progressive correlation coefficient values (R^2^ = 0.95–0.99) than the PFO kinetic model (R^2^ = 0.92–0.98) (Table 2). For 0.1% B−CB, the PSO model provided a better fit than the PFO model, with a higher R^2^ (0.96) and lower RMSE (6.39 mg g^−1^) than the PFO model (R^2^ = 0.93; RMSE = 8.81 mg g^−1^). In particular, the adsorption process on 5% B−CB showed excellent agreement with the PSO kinetic model, yielding the maximum R^2^ value (0.99) along with the lowest RMSE value. Additionally, the equilibrium azithromycin adsorption capacities obtained from the PSO model were reasonably consistent with the experimentally determined values, suggesting that surface interactions may have contributed to the adsorption process. This observation is consistent with the findings of Mouna et al. (2026), who reported that both PFO and PSO kinetic models provided suitable fits to the biosorption data [54].

Among the investigated adsorbents, 5% B−CB showed the greatest adsorption capacity and the fastest adsorption dynamics, as reflected by its higher kinetic rate constants (k_1_ = 0.287 min^−1^ and k_2_ = 3.92 × 10^−3^ g mg^−1^ min^−1^). The improved kinetic performance of 5% B−CB may be associated with the improved number of surface binding sites and enhanced surface reactivity resulting from boric acid modification. Overall, the kinetic analysis confirms that boric acid modification meaningfully improves the adsorption performance of coconut husk−based biochar toward azithromycin removal, with the PSO kinetic model providing the finest explanation of the kinetic mechanism (Figure 7A–C and Table 2).

**Figure 8:**
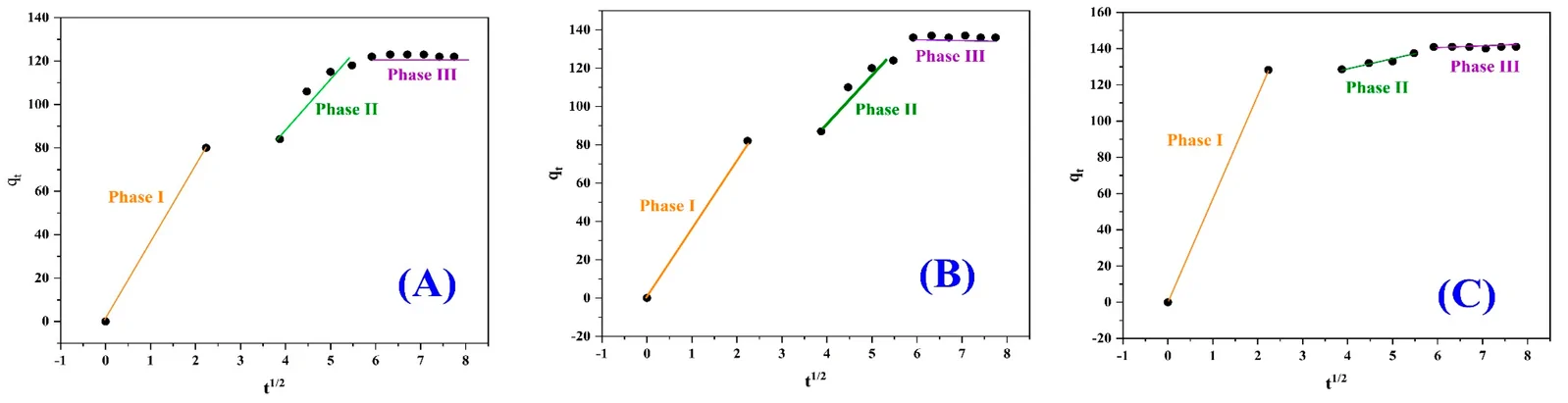
Time−dependent adsorption profile of azithromycin onto (**A**) 0.1% B−CB, (**B**) 2% B−CB, (**C**) 5% B−CB.

Also, the IPD model was utilized to explain the mass transfer behavior of azithromycin adsorption on B−CB. The obtained multilinear diffusion profiles exhibited three distinguishable regions (Figure 8A–C and Table 3), indicating that adsorption proceeded through successive transport phases rather than a single diffusion−controlled mechanism. The preliminary stage was characterized by a steep diffusion gradient, corresponding to the rapid migration of adsorbate species from the solution phase to the readily accessible active sites at the adsorbent interface. This behavior was particularly evident for 5% B−CB, which exhibited the highest first−stage diffusion rate constant (k_id_ = 57.3 mg g^−1^ min^−0.5^), highlighting a comparatively higher apparent contribution of rapid external−to−surface mass transfer under the investigated conditions.

During the second stage, a notable decline in k_id_ values was observed for all adsorbents, suggesting that the biosorption method gradually became regulated by pore diffusion along with transport within the internal structure of the biochar matrix. The reduced diffusion rates are consistent with increasing resistance to azithromycin transport within the internal structure of the biochar matrix [57]. Among the tested adsorbents, 5% B−CB maintained comparatively favorable diffusion characteristics, reflecting comparatively favorable diffusion characteristics under the investigated conditions following H_3_BO_3_−assisted modification.

In the final stage, the diffusion rate constant approached zero, accompanied by substantially higher intercept (C) values. This phenomenon indicates the progressive inundation of adsorption binding sites together with the development of equilibrium state of adsorption. Therefore, the increase in (C) values from the first to the third stage reflects the growing contribution of boundary−layer effects and throughout the adsorption process. Furthermore, no individual linear diffusion plots converged at the origin, demonstrating that IPD did not act as the sole controlling mechanism. Instead, biosorption occurred through the combined influence of boundary−layer diffusion, surface biosorption, combined with intraparticle transport [58,59].

The comparatively higher R^2^ values obtained for the first and second diffusion regions (R^2^ = 0.83–0.99) indicate that the IPD model adequately describes the intermediate transport processes (Table 3, Figure 8). However, the lower correlation coefficients observed in the equilibrium region of 0.1 and 2% B−CB suggest that diffusion became negligible once the available adsorption sites were nearly saturated. Collectively, these findings indicate that the modification conditions were associated with differences in the diffusion dynamics of azithromycin adsorption, with 5% B−CB showing comparatively favorable mass−transfer behavior and rapid initial adsorption. The results indicate that increasing the nominal H_3_BO_3_ precursor concentration was associated with differences in the observed diffusion behavior. However, the specific contribution of boron species to these differences cannot be established from the available characterization data. Therefore, the IPD analysis indicates that azithromycin adsorption onto B−CB involves a complex multi−step transport process, while the comparatively favorable diffusion behavior of 5% B−CB indicates that the H_3_BO_3_−assisted modification conditions were associated with differences in adsorption and mass−transfer behavior. In agreement with López−Luna et al. [60], the deviation of the IPD plots from the origin suggests that adsorption method is involved with a combination of boundary−layer diffusion and intraparticle diffusion [60].

#### 3.3.2. Adsorption Isotherm Models

The azithromycin adsorption equilibrium performance onto (B−CB) was evaluated using the Langmuir, Freundlich, as well as Temkin isotherm models, and the associated fitted curves and calculated parameters are shown in Figure 7D–F and Table 4. The adsorption capacities increased with increasing nominal H_3_BO_3_ precursor concentration, indicating that boron modification improved the affinity of the biochar surface toward azithromycin molecules. Considering the investigated models, the experimental data were well described by the Langmuir isotherm models for all adsorbents, with consistently high coefficients of determination (R^2^ = 0.99) and relatively low RMSE values (2.14–4.98). The corresponding qmax values were (q_max_) 136.92, 158.84, and 170.45 mg g^−1^ for 0.1, 2, and 5% B−CB, correspondingly, signifying a gradual rise in adsorption performance following an increase with increasing nominal H_3_BO_3_ precursor concentration. Furthermore, the dimensionless separation factor (R_L_) spanned the range of 0.15–0.19, remaining between 0 and 1 for all adsorbents, which confirms the favorable nature of azithromycin adsorption onto the modified biochar [61]. Although the Langmuir model is mathematically consistent with a monolayer−type representation, the present equilibrium data alone do not establish a specific monolayer adsorption mechanism. Similar findings were reported by [62], who observed that azithromycin adsorption was significantly defined by the Langmuir isotherm model, reflecting favorable monolayer biosorption behavior [63]. The comparatively better fit of the Langmuir model suggests that the equilibrium data were reasonably represented by a monolayer−type adsorption model under the investigated conditions. The Freundlich isotherm model also satisfactorily defined the biosorption behavior, yielding the values of correlation coefficient (R^2^) including 0.84, 0.87, as well as 0.93 for 0.1% B−CB, 2% B−CB, and 5% B−CB, individually. The Freundlich model constants (K_f_) increased from 27.41 to 38.62 mg g^−1^, representing greater adsorption affinity after H_3_BO_3_−assisted modification. Moreover, the Freundlich intensity parameter (n) ranged from 3.12 to 4.21, suggesting favorable adsorption and a relatively heterogeneous adsorbent surface [64]. However, the comparatively lower R^2^ values indicate that the Freundlich isotherm model was less suitable than the Langmuir model for describing the adsorption equilibrium performance.

Therefore, another isotherm model, the Temkin model, was also evaluated the Temkin model also revealed good association with the investigational data, particularly for 0.1% and 5% B−CB, both showing R^2^ = 0.99 (Figure 7A–C, Table 4). The Temkin constants (B_T_) increased from 16.84 to 20.47 J mol^−1^ with increasing nominal H_3_BO_3_ precursor concentration, indicating differences in the estimated adsorption heat parameter among the modified biochars. Nevertheless, the relatively higher RMSE values obtained for the Temkin model indicate that its empirical fit was inferior to that of the Langmuir model. In line with the current findings, Saadi et al. [28] stated that higher B_T_ values correspond to greater adsorption energy and stronger adsorbent–adsorbate interactions, resulting in improved adsorption performance [28]. Overall, the Langmuir model provided the best statistical description of the equilibrium data among the models evaluated, based on its comparatively high R^2^ values and lower RMSE values. However, the better Langmuir fit should be interpreted as a statistical representation of the equilibrium data and does not, by itself, establish a homogeneous surface or a definitive monolayer adsorption mechanism (Figure 7D–F and Table 4).

#### 3.3.3. Thermodynamic Model

To evaluate the temperature dependence, feasibility, and energetic nature of the adsorption process on 0.1%, 2%, and 5% B−CB composites, standard thermodynamic parameters—including Gibbs free energy change ΔG°, enthalpy change ΔH°, and entropy change ΔS°—were investigated across a temperature range of 298 K to 333 K. To maintain thermodynamic rigor, eliminate potential parameter discrepancies, and ensure internal calculation consistency, two distinct mathematical approaches were employed to determine ΔG°: (i) Direct Equilibrium Calculations ΔG° = −RT lnKL [65,66], which reflect point−by−point experimental thermodynamic states at each discrete temperature, and (ii) Linear Regression Predictions ΔG° = ΔH° − T ΔS°, which capture the global linear trend derived from the Van ’t Hoff plot ln KL vs. 1/T [66]. As presented in Table 5, both computational formulations demonstrated exceptional agreement and yielded nearly identical numerical values across all temperatures and composite ratios—such as 1.045 kJ/mol (direct) vs. 1.087 kJ/mol (predicted) at 298 K for 0.1% B−CB, 0.799 kJ/mol vs. 0.656 kJ/mol for 2% B−CB, and −0.079 kJ/mol vs. −0.172 kJ/mol for 5% B−CB—thereby validating the mathematical integrity and thermodynamic reliability of the underlying equilibrium data. For 0.1% B−CB and 2% B−CB, ΔG° values derived from both formulations transitioned from slightly positive at 298 K to lower or negative values at 333 K −0.132 kJ/mol (direct) vs. −0.085 kJ/mol (predicted) for 0.1% B−CB, indicating that elevated temperatures drive process spontaneity, whereas 5% B−CB exhibited inherently spontaneous negative ΔG° values across all evaluated conditions [67]. Because the distribution coefficient K_L_ = q_e_/C_e_ was expressed in L g^−1^, the calculated ΔG° values are reported within the empirical convention adopted for this adsorption system and should not be interpreted as strictly standardized thermodynamic free energies based on a dimensionless equilibrium constant. Interestingly, the standard enthalpy change (ΔH°) was highest for 0.1% B−CB (11.08 kJ mol^−1^) and decreased for 2% B−CB (6.74 kJ mol^−1^) and 5% B−CB (6.19 kJ mol^−1^). The positive ΔH° values indicate that azithrimycin adsorption was endothermic under the investigated conditions. The relatively higher ΔH° for 0.1% B−CB compared with the modified materials indicates a greater enthalpic contribution to the adsorption process, whereas the lower ΔH° values for 2% and 5% B−CB indicate that less heat was associated with the overall adsorption process under the investigated conditions. These differences may reflect variations in the surface characteristics and adsorption interactions resulting from the H_3_BO_3_−assisted modification [68]. Despite this lower enthalpic heat of adsorption, 5% B−CB exhibited a superior overall adsorption capacity due to a higher density of active surface functional groups and accessible binding sites provided by the greater composite loading, making the process thermally more favorable and energetically efficient. Furthermore, the positive ΔH° values indicate that the adsorption process was endothermic under the investigated conditions. However, the thermodynamic parameters alone do not establish a specific adsorption mechanism, such as physisorption, chemisorption, electrostatic attraction, or van der Waals interactions [8]. Additionally, positive entropy values ΔS° = 33.50, 20.36, and 21.35 Jmol^−1^K^−1^ for 0.1%, 2%, and 5% B−CB, respectively reflect increased randomness at the solid−solution interface due to the displacement of adsorbed water molecules by target pollutants [46,66,68], while the Van’t Hoff relationship showed relatively good linearity for 0.1% B−CB (R^2^ = 0.97) and 5% B−CB (R^2^ = 0.91), whereas the lower R^2^ value for 2% B−CB (0.87) indicates comparatively weaker linearity and warrants cautious interpretation of its thermodynamic parameters [69].

#### 3.3.4. Insights into the Adsorption Mechanism of Azithromycin by 5% B−CB

**Figure 9:**
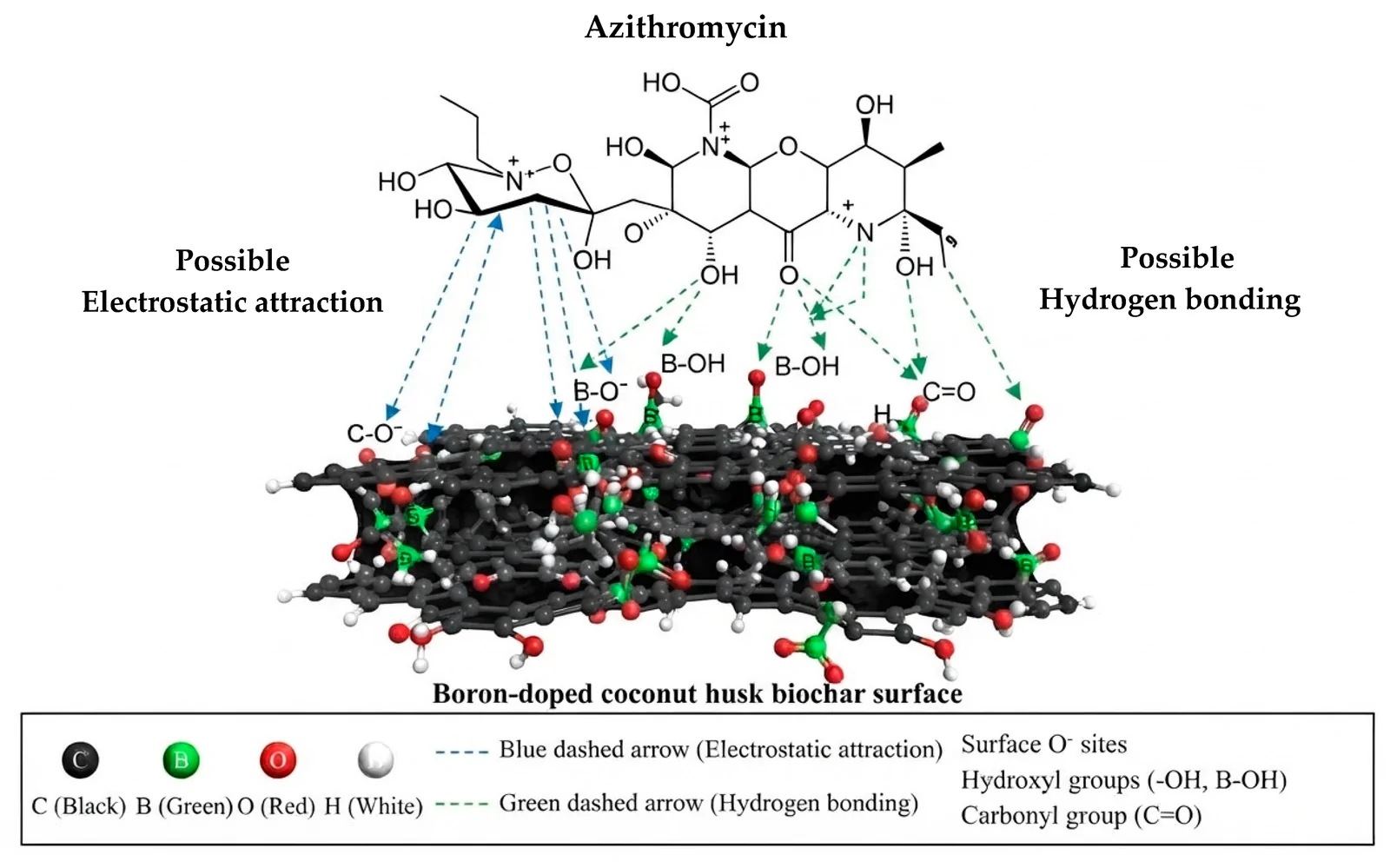
Conceptual schematic of the proposed interactions between azithromycin and 5% B−CB based on the observed adsorption trends and FTIR results.

The azithromycin adsorption examination onto 5% B−CB encompasses numerous physicochemical interactions disposed by both the functional sites on the 5% B−CB and the molecular possessions of azithromycin (Figure 9). Azithromycin is a weakly basic macrolide containing protonatable nitrogen atoms, and based on its reported pKa values, it is expected to occur predominantly in protonated forms at pH 7. The modification−induced incorporation of boron−containing and oxygenated surface functionalities may alter the surface polarity and charge distribution of the biochar, thereby influencing electrostatic interactions with azithromycin. Because azithromycin contains protonatable amine groups, its charge state varies with solution pH, while deprotonated oxygen−containing surface groups can provide negatively charged sites under appropriate conditions. Thus, the enhanced adsorption observed around the favorable pH range may partly reflect electrostatic attraction between protonated azithromycin species and negatively charged surface sites. Because the surface charge of 5% B−CB was not directly measured in the present study, electrostatic attraction between protonated azithromycin and negatively charged surface sites cannot be confirmed. However, if negatively charged oxygen−containing surface groups are present at the experimental pH, electrostatic attraction may contribute to azithromycin adsorption and is therefore considered a possible mechanism [70]. 5% B−CB biochar contains polar surface functional moieties like –OH, –COOH, as well as B–OH introduced or enhanced by boric acid modification. These polar functionalities can enhance hydrogen−bond formation by providing donor and acceptor sites for interaction with the hydroxyl, amine, and ether functionalities of azithromycin. In particular, surface –OH and oxygen−containing groups can participate in hydrogen bonding with heteroatom−containing moieties of the antibiotic, while boron–oxygen functionalities may further modify the polarity and local chemical environment of the adsorption sites. Such interactions can increase the affinity of azithromycin for the modified biochar surface and contribute to the observed adsorption capacity. These functional groups may participate in hydrogen bonding with the hydroxyl, ether, and nitrogen−containing groups of azithromycin. Therefore, hydrogen bonding is considered a plausible contributor to the adsorption process, although it was not directly confirmed in the present study [70]. Boric acid−modified coconut husk biochar surface species may alter the local polarity and electronic environment of the biochar surface and could provide sites capable of interacting with the nitrogen− and oxygen−containing functional groups of azithromycin. Such interactions are therefore considered possible contributors to the overall adsorption process rather than directly verified Lewis acid–base interactions. Accordingly, the interactions illustrated in Figure 9 are intended as conceptual possibilities rather than experimentally confirmed adsorption pathways. Boron−containing surface species may also interact with the electron−donating nitrogen and oxygen atoms of azithromycin through possible Lewis acid–base interactions. However, these interactions were not directly verified by XPS or other surface−sensitive analyses and therefore remain a proposed mechanism. In addition, B–OH groups may participate in hydrogen bonding with hydroxyl, amine, and ether functionalities of azithromycin, while their polar character may further facilitate surface–adsorbate interactions. The combined contribution of these interactions may improve the affinity of azithromycin toward the modified biochar surface. The surface morphology of 5% B−CB may facilitate the accessibility of azihromycin molecules to available adsorption regions. However, because quantitative pore−size distribution and surface−area measurements were not performed, the specific contribution of pore filling to the overall adsorption capacity cannot be independently confirmed. Pore accessibility may therefore contribute to the observed adsorption capacity, particularly as azithromycin molecules gain access to internal adsorption regions during the adsorption process; however, this contribution cannot be independently quantified without quantitative textural characterization [50].

### 3.4. Recyclability Assessment

The current study explores three desorbents such as 50% ethanol (C_2_H_5_OH) in water, 50% ethanol with 0.1 M NaCl, and 50% ethanol with 0.02 M NaOHto evaluate the reusability of 5% B−CB. The use of 50% ethanol may facilitate azithromycin desorption by altering solvent–adsorbate and adsorbate–surface interactions. However, the desorption results alone do not establish the disruption of any specific interaction. Further mechanistic investigations would be required to determine the contributions of hydrogen bonding, electrostatic interactions, and other possible non−covalent interactions during regeneration [71]. The excessive polarity or nonpolarity of pure water or absolute ethanol reduces desorption efficiency or jeopardizes adsorbent stability. By increasing ionic strength, adding sodium chloride to ethanol reduces the electrostatic attraction between anionic biochar surface sites and cationic azithromycin molecules, leading to intermediate desorption [72]. However, NaOH in ethanol creates a mildly alkaline environment that produces the highest desorption efficiency by neutralizing electrostatic interactions, breaking hydrogen bonds, and deprotonating surface functional groups [71].

**Figure 10:**
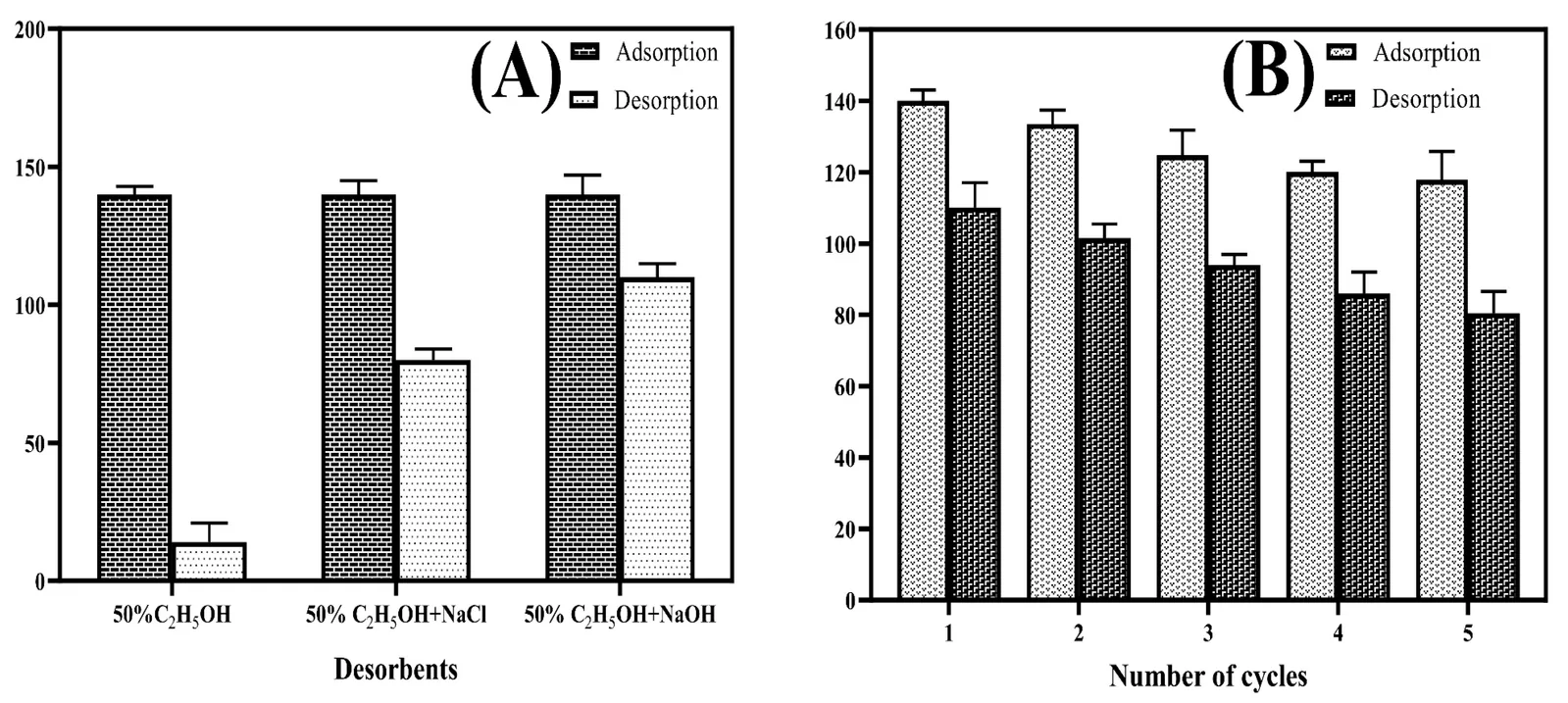
(**A**) Comparison of azithromycin desorption using different eluting solvents from 5% boric acid−modified coconut husk biochar (5% B−CB). (**B**) Adsorption–desorption performance of azithromycin on 5% B−CB over multiple regeneration cycles.

The combined effects of pH and solvent polarity promoted effective regeneration, with 50% ethanol + 0.02 M NaOH exhibiting the highest desorption efficiency (78.6%), corresponding to a desorbed amount of 110 mg g^−1^. This performance was substantially higher than that of 50% ethanol alone (10%, 14 mg g^−1^) and 50% ethanol + 0.1 M NaCl (57.1%, 80 mg g^−1^) (Figure 10). Based on its superior desorption performance, 50% ethanol + 0.02 M NaOH was selected for five consecutive adsorption–desorption cycles at a liquid−to−solid ratio of 100 mL g^−1^ to further assess the reusability of 5% B−CB (Figure 10). A 3% mass loss of the biochar and a desorbed amount of 110.1 mg g^−1^ (78.6% desorption) were observed in the preliminary adsorption performance of 140 mg g^−1^. As desorbed amounts declined from 110.1 to 80.5 mg g^−1^ and adsorption capacity decreased from 140 to 117.9 mg g^−1^ over the course of subsequent cycles, the desorption efficiency declined slightly from 78.6% to 68.3%. The relatively small mass loss observed over the regeneration cycles indicates limited adsorbent mass loss under the tested regeneration conditions. According to these findings, 5% B−CB is efficiently renewed over several cycles using 50% ethanol + NaOH while retaining a significant adsorption capacity and high desorption efficiency. However, the 5% B−CB retained measurable adsorption capacity over five regeneration cycles, although a gradual decline in capacity was observed.

### 3.5. Evaluation of Fixed−Bed Column Performance

**Figure 11:**
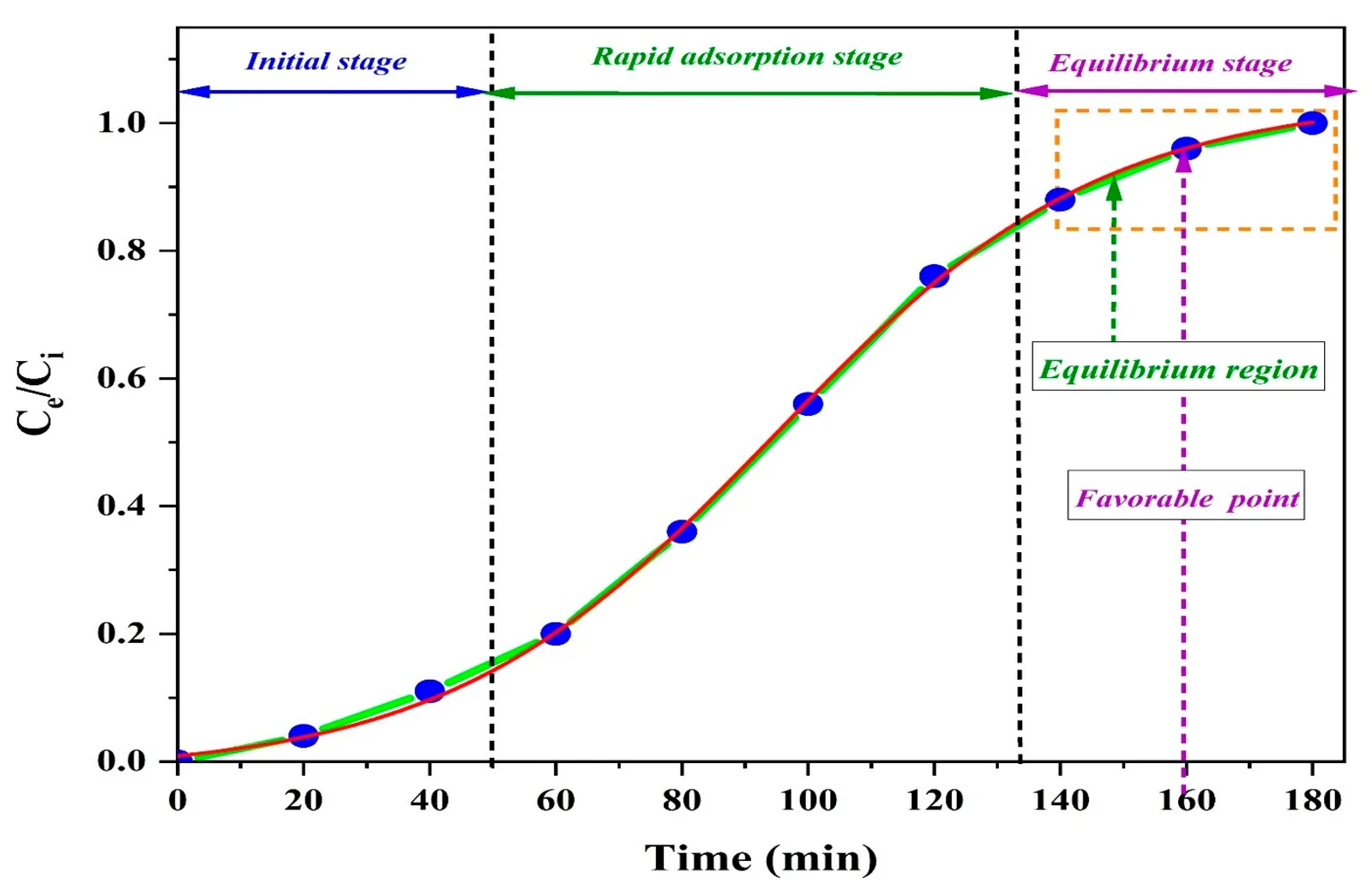
Breakthrough curve of azithromycin adsorption on 5% boric acid−modified coconut husk biochar (5%−B−CB) in a fixed−bed column.

The breakthrough curve of azithromycin adsorption onto 5% B−CB is presented in Figure 11. The normalized effluent concentration (C_t_/C_i_) remained very low during the initial stage of column operation, indicating high initial azithromycin removal by the packed bed. The breakthrough point was defined at C_t_/C_i_ = 0.05 and occurred at approximately 20 min. Thereafter, the effluent azithromycin concentration increased progressively, reaching C_t_/C_i_ values of approximately 0.13 and 0.60 at 40 and 100 min, respectively. The predefined exhaustion criterion of C_t_/C_i_ = 0.95 was reached at approximately 157 min, based on linear interpolation between the experimental points at 140 and 160 min. At 180 min, C_t_/C_i_ reached approximately 1.10, indicating that the effluent concentration had approached and slightly exceeded the influent concentration under the tested operating conditions. The gradual increase in C_t_/C_i_ reflects the progressive development of breakthrough through the packed bed and the associated reduction in azithromycin removal efficiency, consistent with the mass−transfer behavior typically observed in fixed−bed adsorption systems [73]. Importantly, the approach of C_t_/C_i_ toward unity indicates loss of effective removal at the column outlet under the applied conditions and does not by itself demonstrate complete saturation or uniform equilibrium loading of the entire 150 g adsorbent bed. Thus, the observed breakthrough reflects outlet−level exhaustion under the tested operating conditions rather than complete utilization of the entire adsorbent mass.

The dynamic adsorption performance was further evaluated using a mass−balance approach. At an influent azithromycin concentration of 50 mg L^−1^ and a flow rate of 1 mL min^−1^, a total of 180 mL of solution was introduced during the 180 −min experiment, corresponding to 9.00 mg of azithromycin input. The total azithromycin input of 9.00 mg corresponds to an upper−bound loading of 0.060 mg g^−1^ for the 150 g adsorbent bed, assuming complete retention of the influent azithromycin. Numerical integration of the influent–effluent concentration difference up to the defined exhaustion point gave an azithromycin uptake of approximately 4.55 mg, corresponding to a dynamic adsorption capacity of approximately 0.0304 mg g^−1^ for the 150 g adsorbent bed. The corresponding overall removal of azithromycin was approximately 58.1% up to the exhaustion point. These values describe the actual dynamic performance of the packed bed under the applied feed concentration, flow rate, adsorbent mass, and experimental duration.

The Thomas and Yoon–Nelson model parameters and goodness-of-fit statistics are presented in Supplementary Table S1 and Figure S1, with the fixed-bed modeling [74] approach guided by established literature [75,76].

### 3.6. Limitations

The present study has several limitations that should be considered when interpreting the results. First, the actual nominal H_3_BO_3_ precursor concentration of the synthesized biochars was not quantitatively determined using techniques such as ICP−OES, ICP−MS, or XPS. Accordingly, the 0.1%, 2%, and 5% designations represent the nominal concentrations of the H_3_BO_3_ precursor solutions used during modification and should not be interpreted as the actual amount of boron associated with the final biochars of the final biochars. In addition, replicate EDX measurements, quantitative elemental mapping, and detection−limit assessment were not performed. The FTIR and XRD results therefore provide qualitative evidence of changes associated with boric acid−modified coconut husk biochar but cannot quantitatively establish amount of boron associated with the final biochars or its spatial distribution. Second, quantitative textural characterization by BET analysis and direct surface−charge characterization through pHpzc and zeta−potential measurements were not performed. Consequently, the contributions of specific surface area, pore structure, surface charge, and electrostatic interactions to azithromycin adsorption cannot be quantitatively resolved from the present dataset. The mechanistic interpretations presented in this study are therefore limited to plausible explanations based on the observed adsorption trends and spectroscopic characterization rather than definitive identification of the underlying adsorption mechanism. Future studies should incorporate quantitative elemental and surface−chemical characterization, including ICP−OES/ICP−MS, XPS, replicated EDX with elemental mapping, BET surface−area and pore analysis, and pHpzc/zeta−potential measurements. Such complementary analyses would help clarify the relationship between actual H_3_BO_3_ precursor level, surface properties, adsorption behavior, and the underlying interaction mechanisms. Finally, the fixed−bed experiment was conducted with a relatively large adsorbent mass (150 g) compared with the total azithromycin mass introduced during the 180 min run (9.00 mg). Consequently, the bed was not fully utilized, and the calculated dynamic capacity represents the uptake achieved under the tested operating conditions rather than a fully developed bed capacity. Future column studies should optimize adsorbent mass, influent concentration, flow rate, and operation time to achieve greater bed utilization and enable more representative dynamic−capacity assessment.

## 4. Conclusions

This study demonstrated the potential of boric acid−modified coconut husk biochar (B−CB) for the removal of azithromycin from aqueous media. The adsorbents prepared using nominal H_3_BO_3_ precursor concentrations of 0.1%, 2%, and 5% exhibited maximum experimental adsorption capacities of 121, 136.1, and 141 mg g^−1^, respectively, under the optimized experimental conditions. Among the tested materials, the 5% B−CB exhibited the highest adsorption capacity. Adsorption performance was influenced by the initial azithromycin concentration, adsorbent dosage, solution pH, contact time, and temperature.

FTIR, FESEM, EDX, and XRD analyses provided qualitative information on the functional groups, surface morphology, elemental composition, and structural characteristics of the prepared materials. The observed changes in the FTIR spectra and surface morphology following boric acid modification suggest alterations in the surface characteristics of the biochar. However, because BET surface−area, pore−volume, pHpzc, and zeta−potential measurements were not performed, the quantitative contributions of surface area, pore structure, and surface charge to azithromycin adsorption could not be established. Therefore, the observed characterization results should not be considered definitive evidence of a specific molecular adsorption mechanism.

The kinetic data were better described by the pseudo−second−order model than by the pseudo−first−order model under the investigated conditions. Thus, the pseudo−second−order equation provided a better mathematical description of the adsorption kinetics; however, kinetic−model fitting alone does not establish chemisorption or a specific molecular−level adsorption mechanism. The equilibrium data were evaluated using the Langmuir and Freundlich models to describe the adsorption behavior of azithromycin on B−CB. The experimentally determined equilibrium adsorption capacities were approximately 121, 136.1, and 141 mg g^−1^ for the 0.1%, 2%, and 5% B−CB, respectively. The isotherm model providing the better statistical fit was considered the more appropriate empirical description of the equilibrium data, without interpreting the model assumptions as definitive evidence of the actual molecular adsorption configuration.

Thermodynamic analysis indicated favorable adsorption within the investigated temperature range, as reflected by the negative ΔG° values, while the positive ΔH° value suggested an endothermic adsorption process under the experimental conditions. These thermodynamic findings should be interpreted within the scope of the investigated aqueous system and temperature range.

The fixed−bed experiment demonstrated breakthrough behavior and measurable azithromycin uptake under the investigated operating conditions. However, the relatively low dynamic loading should be interpreted in relation to the experimental column conditions and the limited contaminant mass introduced relative to the adsorbent bed, rather than as the full utilization capacity of the adsorbent.

Overall, the results indicate that boric acid−modified coconut husk biochar has potential for azithromycin removal from aqueous solutions. Further research should focus on regeneration and reuse, quantitative surface−area and pore−property characterization, surface−charge characterization, adsorption in complex and real wastewater matrices, identification of the specific interactions potentially involved in azithromycin uptake, and evaluation under continuous−flow conditions. Scale−up studies using the material prepared with the highest nominal H_3_BO_3_ precursor concentration (5% B−CB) may also be considered for future investigation.

## Figures and Tables

**Table 2:** Adsorption kinetics and rate constants for azithromycin on 0.1% B−CB, 2% B−CB and 5% B−CB.

Adsorbents	Experimental Data		Kinetic Model Constants							
	Initial Conc. (mgL^−1^)	q_e (exp)_ (mg g^−1^)	PFO				PSO			
			q_e_ (Cal) (mg g^−1^)	K_1_ (min^−1^)	R^2^	RMSE	q_e_ (Cal) (mg g^−1^)	K_2_ (g mg^−1^ min^−1^)	R^2^	RMSE
0.1% B−CB	250	121	119.75	0.144	0.93	6.39	132.44	1.74 × 10^−3^	0.96	8.81
2% B−CB	250	136.1	133.62	0.123	0.92	8.07	148.91	1.18 × 10^−3^	0.95	5.11
5% B−CB	250	141	140.87	0.287	0.98	2.14	146.32	3.92 × 10^−3^	0.99	1.03

**Table 3:** Intraparticle diffusion (IPD) model considerations for azithromycin adsorption onto 0.1% B−CB, 2% B−CB and 5% B−CB.

Adsorbent	Phase	k_id_ (mg g^−1^ min^−1/2^)	C (mg g^−1^)	R^2^
0.1% B−CB	Phase I	22.1	3.8	0.83
	Phase II	20.8	29	0.96
	Phase III	−0.4	124.9	0.44
2% B−CB	Phase I	23.30	8.88	0.86
	Phase II	22. 23	5.43	0.94
	Phase III	−0.14	137.31	0.035
5% B−CB	Phase I	57.3	0	0.99
	Phase II	8.7	92.8	0.97
	Phase III	0.41	138	0.99

**Table 4:** Isotherm modeling and parameters for azithromycin adsorption onto 0.1% B−CB, 2% B−CB and 5% B−CB.

Adsorbents	Langmuir Parameters					Freundlich Parameters				Temkin Parameters			
	K_L_ (L mg^−1^)	R_L_	q_max_ (mg g^−1^)	R^2^	RMSE	K_f_ (mg g^−1^)	n	R^2^	RMSE	K_T_ (L mg^−1^)	B_T_ (J mol^−1^)	R^2^	RMSE
0.1% B−CB	0.0118	0.195	136.92	0.99	4.98	27.41	3.12	0.84	7.36	0.58	16.84	0.99	8.41
2% B−CB	0.0152	0.158	158.84	0.99	2.86	31.74	3.88	0.87	6.14	0.74	18.96	0.98	2.86
5% B−CB	0.0187	0.176	170.45	0.99	2.14	38.62	4.21	0.93	5.87	0.91	20.47	0.99	7.12

**Table 5:** Evaluation of thermodynamic parameters for azithromycin adsorption onto 5% B−CB.

Adsorbents	T (K)	ΔG° = −RTlnK_L_ (kJ mol^−1^)	ΔG° = ΔH° − T ΔS° (kJ mol^−1^)	ΔH° (kJ mol^−1^)	ΔS° (Jmol^−1^K^−1^)	R^2^
0.1% B−CB	298	1.045	1.087	11.08	33.50	0.97
	308	0.868	0.752			
	318	0.338	0.417			
	328	0.174	0.082			
	333	−0.132	−0.085			
2% B−CB	298	0.799	0.656	6.74	20.36	0.87
	308	0.328	0.453			
	318	0.169	0.250			
	328	0.087	0.048			
	333	0.044	−0.053			
5% B−CB	298	−0.079	−0.172	6.19	21.35	0.91
	308	−0.493	−0.385			
	318	−0.680	−0.599			
	328	−0.746	−0.812			
	333	−0.893	−0.919			

## Data Availability

The raw data supporting the conclusions of this article will be made available by the authors on request.

## Supplementary Materials

The following supporting information can be downloaded at https://www.mdpi.com/article/10.3390/toxics14100927/s1, Figure S1: Van ‘t Hoff thermodynamic plots evaluating the adsorption parameters for (A) 0.1% B-CB, (B) 2% B-CB, and (C) 5% B-CB; Table S1: Parameters obtained from Thomas and Yoon–Nelson models for azithromycin adsorption in the fixed-bed column.

## Abbreviations

The following abbreviations are used in this manuscript:

P−CB	Pristine coconut husk biochar
Qmax	Maximum adsorption capacity (mg g^−1^)
0.1% B−CB	0.1% boric acid−modified coconut husk biochar
RL	Langmuir constant (L/mg)
2% B−CB	2% boric acid−modified coconut husk biochar
Kf	Freundlich constant (L/g), constant of sorption
5% B−CB	5% boric acid−modified coconut husk biochar
T	Absolute temperature (K)
q	Adsorption capacity (mg g^−1^)
R	Universal gas constant (8.314 J mol^−1^ K^−1^)
V	Azithromycin solution volume (L)
ΔS°	Entropy (J mol^−1^ K^−1^)
m	Adsorbent weight (g)
ΔH°	Enthalpy (kJ mol^−1^)
C_i_	Initial concentration (mg L^−1^)
ΔG°	Gibbs free energy change (kJ mol^−1^)
Cƒ	Final concentration (mg L^−1^)
RMSE	Root mean square error
C_e_	Equilibrium concentration (mg L^−1^)
q_i_,exp	Experimentally determined adsorption capacity (mg g^−1^)
q_t_	Adsorption capacity at time t (mg g^−1^)
q_i_,cal	Calculated adsorption capacity (mg g^−1^)
q_e_	Adsorption capacity at equilibrium (mg g^−1^)
K_1_	Pseudo−first−order rate constant (min^−1^)
K_2_	Pseudo−second−order rate constant (g mg^−1^ min^−1^)
