# Supplementary material for: Effective Removal of Azithromycin from Aqueous Solution Using Coconut Husk Derived Boric Acid−Modified Biochar
Source: Toxics. 2026 Oct 20;14(10):927. doi: 10.3390/toxics14100927 (PMC13709760; doi:10.3390/toxics14100927)
Supplement: Supplementary file 1 [file toxics-14-00927-s001.zip › toxics-4537260-supplementary.pdf]

2.5. Packed-bed column adsorption experiments

The breakthrough behavior was evaluated by plotting the normalized effluent concentration (C_e/C_i) against the operating time. The azithromycin removal percentage at each sampling time was calculated as

$$Removal\ (\%) = (1 - \frac{C_t}{C_i}) \times 100 \qquad (S1)$$

The cumulative amount of azithromycin adsorbed by the packed bed was determined by numerical integration of the concentration difference between the influent and effluent streams[1]:

$$M_{ads} = Q \int_0^{t_e} (C_i - C_t) dt \qquad (S2)$$

where Q is the volumetric flow rate (L min^{-1}), C_i is the influent azithromycin concentration (mg L^{-1}), C_t is the effluent azithromycin concentration at time t (mg L^{-1}), and t_e is the exhaustion time (min). Numerical integration was performed using the trapezoidal rule based on the experimentally measured effluent concentrations. The dynamic adsorption capacity was calculated as

$$q_{dyn} = \frac{M_{ads}}{M} \qquad (S3)$$

where M is the mass of adsorbent packed in the column (g).

The breakthrough capacity q_b was calculated from the cumulative amount of azithromycin adsorbed up to the breakthrough point:

$$q_b = \frac{M_{ads,b}}{M} \qquad (S4)$$

Where Mads,b is the cumulative amount of azithromycin adsorbed up to the breakthrough time tb. Breakthrough was defined at Ct/Ci=0.05, whereas exhaustion was defined at Ct/Ci=0.95.

The exhaustion capacity q_e was calculated as

$$q_e = \frac{M_{ads,e}}{M} \qquad (S5)$$

where $M_{ads,e}$ is the cumulative amount of azithromycin adsorbed up to the exhaustion time t_e.

The mass-transfer-zone (MTZ) length was estimated using

$$L_{MTZ} = Z(1 - \frac{t_b}{t_{ie}}) \times 100 \qquad (S6)$$

where Z is the packed-bed height (cm), t_b is the breakthrough time (min), and t_e is the exhaustion time (min).

The experimental breakthrough data were further analyzed using the Thomas and Yoon–Nelson models. The linear form of the Thomas model was expressed as follows [1,2]:

$$ln\left(\frac{C_t}{C_i}-1\right)=\frac{K_{Th}q_0M}{Q}-K_{Th}C_it \qquad (S7)$$

where K_{Th} is the Thomas rate constant (L mg^{-1} min^{-1}), q_0 is the theoretical maximum column adsorption capacity (mg g^{-1}), M is the adsorbent mass g, Q is the flow rate (L min^{-1}), and t is the operating time (min). Linear regression of $ln\left(\frac{C_t}{C_i}-1\right)$ versus t was used to estimate K_{Th} and q_0.

The Yoon–Nelson model was expressed as follows [1,2]:

$$ln\left(\frac{C_t}{C_i-C_t}\right)=K_{YN}(t-\tau) \qquad (S8)$$

where K_{YN} is the Yoon–Nelson rate constant (min^{-1}), and τ is the time required for 50% azithromycin breakthrough C_t/C_i=0.50. The model parameters were obtained by linear regression.

3.5. Evaluation of fixed-bed column performance

The dynamic adsorption performance of 5% B-CB toward azithromycin was evaluated using a fixed-bed column containing 150 g of adsorbent at a constant flow rate of 1 mL min^{-1}. The breakthrough curve, expressed as C_t/C_i versus operating time, showed a gradual increase in the normalized effluent concentration with increasing column operation time. Initially, the effluent AZM concentration was very low, indicating effective retention of azithromycin by the packed bed. The normalized concentration increased from 0.04 at 20 min to 0.11 at 40 min, followed by a more pronounced increase to 0.56 at 100 min and 0.88 at 140 min. The concentration reached 0.96 at 160 min, indicating that the adsorption sites within the bed were approaching exhaustion.

Using C_t/C_i=0.05 as the breakthrough criterion, the breakthrough time was estimated by linear interpolation to be approximately 22.9 min. The exhaustion time, defined at C_t/C_i=0.95, was approximately 157.5 min. Thus, the column provided effective azithromycin removal during the initial stage of operation, followed by a progressive reduction in removal efficiency as the adsorption sites became saturated. At the exhaustion point, the azithromycin removal efficiency was approximately 5%, whereas the initial removal efficiency was close to 100%.

The cumulative adsorption performance was determined by numerical integration of the concentration difference between the influent and effluent streams up to the exhaustion point. For the normalized breakthrough data, the integrated adsorption area was approximately 92.09 min. Therefore, the cumulative azithromycin uptake can be expressed as M_{ads}=0.09209C_i mg for a flow rate of 1 mL min^{-1}, where C_i is expressed in mg L^{-1}. For example, at an influent concentration of 50 mg L^{-1}, approximately 4.60 mg of azithromycin was adsorbed up to the defined exhaustion

point, corresponding to a dynamic adsorption capacity of approximately 0.0307 mg g^{-1} for the 150 g packed bed. The corresponding breakthrough capacity was approximately 0.0039 mg g^{-1}.

The estimated mass-transfer-zone length was approximately 21.7 cm, calculated from the breakthrough and exhaustion times and the total bed height of 25.4 cm. The relatively large MTZ compared with the total bed height indicates that mass transfer occurred over a substantial portion of the packed bed rather than being confined to a narrow adsorption front. The progressive increase in C_t/C_i=0.95 further suggests gradual movement of the adsorption front through the column.

Overall, the column experiment demonstrated effective initial azithromycin removal by 5% B-CB, followed by gradual breakthrough and eventual exhaustion of the packed bed. However, the dynamic adsorption capacity obtained from the present column conditions was substantially lower than the equilibrium adsorption capacity obtained from batch experiments. This difference is attributable to the short hydraulic residence/contact time, low influent loading, incomplete utilization of the total adsorbent bed before the defined exhaustion point, and the hydrodynamic and mass-transfer limitations inherent to continuous-flow operation[3]. Therefore, the column results should be interpreted as a demonstration of breakthrough behavior rather than as evidence that the full equilibrium adsorption capacity of the adsorbent was utilized under the applied operating conditions [4].

Table S1. Parameters obtained from Thomas and Yoon–Nelson models for azithromycin adsorption in the fixed-bed column.

**Model**	**k**	**q_0 / τ**	**R^2**	**RMSE**
**Thomas**	8.58×10^{-4} L mg^{-1} min^{-1}	155.6 mg g^{-1}	0.9989	0.01255
**Yoon–Nelson**	0.04366 min^{-1}	91.91 min	0.9983	0.01390

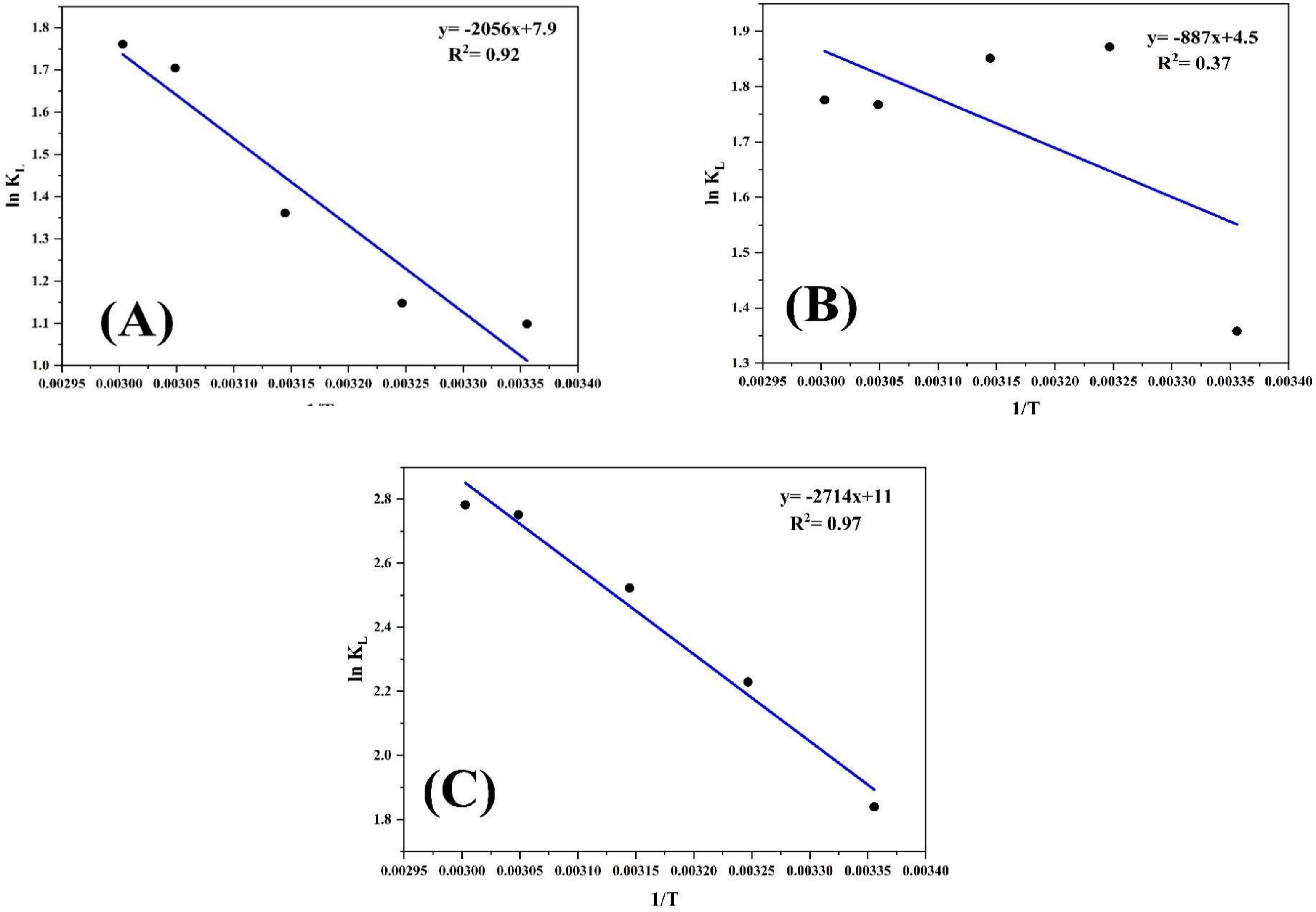

Figure S1. *Van 't Hoff thermodynamic plots evaluating the adsorption parameters for (A) 0.1% B-CB, (B) 2% B-CB, and (C) 5% B-CB.*

References

1. Chu, K.H. Breakthrough Curve Analysis by Simplistic Models of Fixed Bed Adsorption: In Defense of the Century-Old Bohart-Adams Model. *Chem. Eng. J.* **2020**, *380*, 122513.

2. Chu, K.H. Fixed Bed Sorption: Setting the Record Straight on the Bohart–Adams and Thomas Models. *J. Hazard. Mater.* **2010**, *177*, 1006–1012.

3. Patel, H. Fixed-Bed Column Adsorption Study: A Comprehensive Review. *Appl. Water Sci.* **2019**, *9*, 45.

4. Apiratikul, R.; Chu, K.H. Improved Fixed Bed Models for Correlating Asymmetric Adsorption Breakthrough Curves. *J. Water Process Eng.* **2021**, *40*, 101810.
